# Seasonal Dynamics of Foliar Fungi Associated with the Invasive Plant *Ageratina adenophora*

**DOI:** 10.3390/microorganisms14010084

**Published:** 2025-12-30

**Authors:** Yu-Xuan Li, Ai-Ling Yang, Xiao-Han Jin, Zi-Qing Liu, Yong-Lan Wang, Chao Zhao, Zhao-Ying Zeng, Yu-Peng Geng, Han-Bo Zhang

**Affiliations:** 1 State Key Laboratory for Conservation and Utilization of Bio-Resources in Yunnan, Yunnan University, Kunming 650500, China; 22022130033@mail.ynu.edu.cn (Y.-X.L.);; 2 School of Ecology and Environmental Science, Yunnan University, Kunming 650500, China; 3 Faculty of Life Science and Technology, Kunming University of Science and Technology, Kunming 650500, China; 4 State Key Laboratory for Vegetation Structure, Function and Construction (VegLab), Yunnan Key Laboratory of Plant Reproductive Adaptation and Evolutionary Ecology, Yunnan University, Kunming 650500, China

**Keywords:** *Ageratina adenophora*, foliar endophyte, pathogenic fungi, seasonal dynamics, transmission mode

## Abstract

The potential of invasive plants to serve as reservoirs for plant pathogenic fungi has been confirmed, but studies examining the seasonal effects on the community structure and transmission patterns of leaf pathogens within invasive plant populations remain scarce. In this study, we characterised the seasonal dynamics of pathogenic fungal communities in the leaf tissue of the invasive plant *A. adenophora* via culture-dependent and culture-independent methods. The study confirmed that fresh leaves of *A. adenophora* accumulate diverse pathogenic fungi, including Colletotrichum, Epicoccum, Toxicocladosporium, Mycosphaerella and Didymella. These genera are globally distributed and act as pathogens for a wide range of wild plants and economic crops. The pathogenic fungal communities exhibited seasonal dynamics, though the magnitude of change was less pronounced than that of the overall fungal community. Among four common environmental factors, namely, temperature, relative humidity, wind speed and precipitation, temperature had a greater effect on the overall community than the other environmental factors, whereas precipitation had the least effect. However, relative humidity has the strongest effect on the pathogenic fungal community; moreover, relative humidity distinctively affects the same species occurring in different microenvironments. Most foliar pathogenic fungi are actively transmitted in spring and autumn, and very few genera can transmit across all seasons. Moreover, most fungal genera can transmit from fresh leaves to dead leaves, suggesting that most foliar fungal pathogens associated with *A. adenophora* are likely necrotrophic. Our study strongly confirms the potential of the invasive plant *A. adenophora* to act as a reservoir of pathogenic fungi and provides preliminary insights into their potential transmission patterns. These findings underscore that, under suitable climatic and environmental conditions, *A. adenophora* may pose a latent risk of triggering disease transmission within ecosystems.

## 1. Introduction

In the field of invasion ecology, the role of pathogenic microorganisms in the transmission and spread of invasive plants has garnered increasing attention [1,2]. Some have suggested that exotic plants invade successfully because they escape attack by natural enemies, especially fungal pathogens, i.e., the enemy release hypothesis [3]. For example, a study of 473 plant species introduced into the United States from Europe revealed that the number of fungal and viral species infecting each plant species within the introduced range was reduced by an average of 84% and 24%, respectively, compared with their native range [4]. Research on the temperate invasive plant *Ambrosia trifida* has revealed that its seed germination rate, plant growth rate, and population density are significantly higher in nonnative soils than in native soils. This is precisely because it escapes the presence of more underground pathogen natural enemies [5]. However, some studies have also shown that the escape of pathogens by exotic plants is only a temporary phenomenon, and with increasing invasion time and geographical distribution range, many exotic plants accumulate local plant pathogens to varying degrees in the process of invading new habitats, i.e., the hypothesis of accumulation of local pathogens (ALP) [6]. The ALP suggests that pathogens accumulated by invasive alien plants may spread to native plants through “spillover” and “spillback”, producing a competitive disadvantage, whereas exotic species become more tolerant to the pathogen than native ones [7,8] (but also see [9]). For instance, the invasion of the alien plant *Chromolaena odorata* has significantly increased the density of the native plant pathogen (*Fusarium*) within the Western Ghats ecosystem of India, leading to a reduction in the diversity of surrounding native flora [10]. Similarly, the colonisation of the invasive plant *Carpobrotus eduliss* significantly increased the abundance of native plant pathogens (Chytridiales), leading to a decline in plant diversity along the Mediterranean coast [11]. This competitive disadvantage is primarily attributable to the fact that invasive species often exhibit greater tolerance to pathogens than native species. Research indicates that predation pressure reshapes the defence strategies of invasive plants, redirecting defensive resources towards growth to facilitate invasion [12]. Furthermore, invasive plants demonstrate enhanced adaptability to environmental stressors, leveraging such stresses to bolster their resistance to disease. Research indicates that nitrogen deposition and heavy metal contamination can enhance the disease resistance of invasive plants [13,14]. Regarding their subterranean components, invasive plants establish more efficient mutualistic relationships with rhizosphere microorganisms—particularly arbuscular mycorrhizal fungi—thereby promoting their own nutrient uptake, growth and development, and disease resistance [15,16]. Regardless of whether invasive plants can increase their advantage when competing with native plants through interactions with pathogens, it is expected that the disease risk driven by plant invasions is a common phenomenon. Therefore, characterising pathogen transmission driven by invasive plants in invaded ecosystems is necessary.

The phyllosphere refers broadly to the aboveground parts of terrestrial plants, including the leaves, flowers, stems, and fruits. As important components of ecosystems, plant phyllospheres provide complex and diverse habitats and ecological niches for epiphytic and endophytic microorganisms [17]. In turn, these phyllosphere microbes affect plants positively or negatively. For example, phyllosphere microbes can promote plant growth, help plants resist disease and adapt to climate change [18,19]; phyllosphere microbes also increase plant tolerance to a wide range of environmental stresses, including temperature extremes, UV irradiation, and drought stress [20]. Nonetheless, phyllosphere microorganisms can negatively affect plants, for example, by competing with them for water and nutrients or by becoming plant pathogens and causing disease damage to plants [21,22]. Compared with the rhizosphere, the phyllosphere serves as a more open system with an associated microbiome that acts as a reservoir for microbial transmission and storage, with two main modes of horizontal transmission (surrounding plants or the environment) and vertical transmission (by seeds) [23]. Thus, phyllosphere-associated phytopathogenic fungi are expected to increase the invasiveness of invasive plants through a variety of actions.

At present, research on air-plant pathogenic microorganisms has focused mainly on the disease triangle, a research model proposed by Russell B. Stevens in 1960. This model broadly defines the parameters of the disease triangle as “the inherent susceptibility of the host, the inoculative potential of the host organism, and the influence of the environment on the host organism and pathogenesis” [24]. Plant canopy air with microclimates is an important space for dynamic host-interactions, where pathogens in the canopy air can infect plants under optimal microclimatic conditions (e.g., appropriate temperature and humidity) and in turn release their spores into the air to complete their life cycle. Moreover, hosts can modify the microclimate of the vegetation canopy to influence pathogen transmission and disease development [25]. Most leaf endophytes are spread to host plants via the horizontal transmission of airborne spores, which occurs via the epiphytic germination of propagules, followed by penetration through the cuticle or entry through stomata, with the accumulation of many infections in leaves soon after germination [26]. However, when the leaf surface is wet, some of these endophytes continue to enter the leaf tissue of other plants via horizontal transmission and persist as endophytes without causing detectable damage [27].

Interestingly, for the leaf endophyte-dead leaf system, it is currently believed that there is a high overlap between the leaf-endophyte fungi and the fungi of the dead leaves, whereas with the dying of the plant leaves, some fungi are capable of surviving in the soil and may revert back to the plant by infecting a new host through mycelium, and some other saprophytic fungi are capable of biotrophic exchanges with the host plant in the fresh leaves [28,29,30]. Some species can only survive in the apoplast for a short period of time and can complete their life cycle by rapidly recolonising the living leaves of a new host plant [31]; however, many fungi possess enzyme systems that degrade complex structural plant compounds, such as cellulose and lignin, and some of these enzymes may contribute to the invasion of new plant hosts [32]. Consequently, accumulated leaf pathogens from invasive plants may spread to surrounding plants through the above pathways under the right conditions, creating a potential risk of plant disease spread in the invaded ecosystem.

Owing to the complexity and openness of the phyllosphere environment, the phyllosphere microbiome is diverse in origin and continues to be influenced by a variety of environmental factors, resulting in distinct seasonal dynamics [33]. Both temperature and humidity can significantly affect the distribution, multiplication and spread of plant pathogens [34,35,36]. For example, elevated temperatures negatively affect potential fungal pathogens [37]. Moisture availability (rainfall and humidity) has also been shown to be significantly correlated with the occurrence of plant diseases and to predict changes in the abundance of fungal pathogens [38,39]. The phyllosphere microbiome and plant disease levels are always dynamic under the constant influence of seasonal dynamics [40]. However, the effects of seasonality on the community structure and transmission patterns of foliar pathogenic fungi of invasive plant species have not yet been revealed.

*Ageratina adenophora* is an invasive herbaceous perennial plant of the genus *Ageratina* in the Asteraceae family that originated in Mexico and is widely distributed in 40 countries, including China [41]. In the 1940s, the plant was introduced from Myanmar to Yunnan Province in China and spread rapidly, causing ecological problems in southwestern China and becoming one of the most serious invasive alien species in the country [42]. *A. adenophora* harbours diverse leaf fungal endophytes and pathogens [43,44]; in particular, the pathogen community of native plants shares some extent with *A. adenophora* leaf spot pathogens or leaf endophytes, including *Passalora*, *Baeodromus*, *Phaeoramularia* [45], *Alternaria* [46], *Colletotrichum* [47], *Didymella* [48], *Epicoccum* [44] and other fungal pathogens. The potential of *A. adenophora* as a reservoir of phytopathogenic fungi has been demonstrated, and invasion can create a potential risk of ecological harm. Therefore, understanding the effects of seasonality on the community structure and transmission patterns of foliar pathogenic fungi of *A. adenophora* is important.

In this study, the canopy air and leaf tissues of the *A. adenophora* population located in Yunnan Province, China, were continuously collected each month for one year. We analysed the fungal community composition via isolation culture method (hereafter referred to as ICM) and culture-free method (hereafter referred to as CFM). The aims of this study were to investigate the structure and seasonal dynamics of foliar fungi and canopy airborne fungal communities in *A. adenophora* populations. We propose the following hypotheses: (i) Pathogenic fungi are susceptible to temperature and humidity constraints. During suitable seasons, reduced environmental stress promotes their growth and spread. Consequently, the diversity of pathogenic fungal communities peaks in summer and autumn. (ii) The physical stability of leaf litter determines its buffering capacity against external climatic fluctuations. Thus, compared to air and litter, the seasonal structural changes in endophytic fungal communities within fresh leaves are smaller. (iii) The production and activity of pathogen dispersal structures (e.g., spores) exhibit seasonal dependence and are directly regulated by climatic factors. Consequently, the phenomenon of endophytic fungal dispersal within leaves is more pronounced during environmentally favourable warm and humid seasons and is also associated with specific taxonomic groups.

## 2. Materials and Methods

### 2.1. Description of Research Site

Leaf tissue and canopy air samples from plants were collected monthly from three fixed plots, all of which were located in Xishan Forest Park, Yunnan Province, China; these plots belong to the northern latitude subtropical highland mountain monsoon climate and have an average elevation of 1995 m (specific locations: N 24°58’11″ E 102°37’49″ 1956 m; N 24°58’7″ E 102°37’46″ 2051 m; N 24°58’26″ E 102°37’49″ 1979 m). The forest coverage of the area is 94%, and it is an excursion scenic area characterised mainly by scenic forests. The vegetation types mainly include warm-temperate coniferous forests (e.g., *Pinus yunnanensis*, *Pinus armandii*, *Keteleeria evelyniana*), deciduous broadleaf forests (*Alnus nepalensis*), and semimoist evergreen broadleaf forests (*Cyclobalanopsis glaucoides*, *Castanopsis delavayi*) [44]. There are multiple invasion-forming monodominant communities of *A. adenophora* in the forest understorey. In addition, environmental data were obtained from the nearest environmental monitoring station for each sampling month (Table S1)).

### 2.2. Sample Collection

From October 2019 to April 2021, fresh leaves, dead leaves, and canopy air samples from the invasive plant *A. adenophora* were collected from each plot. Owing to the impact of COVID-19 control in early 2020, the sampling, which was originally scheduled for January to April 2020, was conducted from January to April 2021. For each sampling, we selected a sunny day at the end of each month for sampling. The feasibility of using air sampling to investigate the diversity and seasonal dynamics of airborne fungi has been demonstrated on the basis of a recent study [49]. Since this method captures airborne fungal spores, it is possible to characterise fungal colonisation and dispersal with high temporal resolution [49]. Here, air sampling was performed via the SAS Super ISO 180 Microbial Air Sampler (VWR, Radnor, PA, USA). The instrument was placed at the height of the plant canopy in the *A. adenophora* population (approximately 5–10 cm above the tallest leaves). A Petri dish containing sterile cellophane was placed into the instrument, and 1000 L of air was extracted to attach the fungal spores to the cellophane. After completion, the cellophane was removed, placed in a sterile zip-lock bag and returned to the laboratory for storage at −80 °C. In addition, 1/5 the concentration of PDA medium supplemented with antibiotics (streptomycin sulfate and ampicillin sodium salt, 40 µg/mL and 20 U/mL) was added to the instrument, 100 L of air was extracted, and the fungal spores were attached to the medium. After completion, the Petri dish was sealed and returned to the laboratory for cultivation in an incubator at 28 °C. Three cellophane samples and three culture medium samples were collected from each plot, with the machine’s position being adjusted for each collection. The desired leaf tissue is collected from the same population where the air is collected. Three healthy plants from each population were selected, the fourth pair of healthy leaves and the following pair of dead leaves from each plant were collected, placed into sterile zip-lock bags, labelled and sent back to the laboratory, and the samples were processed within 24 h. Five to seven healthy plants were selected from the population, and their fourth pair of fresh leaves and any undropped senescent leaves were collected from the plant’s lower sections. These samples were placed in sterile sealed bags, labelled, and returned to the laboratory. Subsequent processing of the leaf samples must be completed within 24 h.

### 2.3. Isolation and Culture of Fungi and DNA Extraction

The culture of airborne fungi involved removing the PDA medium with air spores from the incubator, adding the emerging colonies to the new medium for culture purification, and counting. Leaf tissue fungi were isolated and cultured according to the method described by Arnold and Lutzoni [50]. After the leaves were rinsed with running water, the surface was disinfected (soaked in 0.5% sodium hypochlorite solution for 2 min, soaked in 75% alcohol for 2 min, and rinsed in sterile water 3 times), and each leaf was cut in half along the central vein. Half of the leaf tissue from each sample was placed in a sterile glycerol tube and stored at −80 °C. The other half was cut into tissue blocks of similar shape and size (approximately 2 × 2 mm), affixed with sterile tweezers at 1/5 the concentration of potato dextrose agar (PDA) medium, and sealed in an incubator at 28 °C for 3–5 days. When mycelia were observed on the edge of the leaf tissue, new PDA medium was selected for culture purification. All fungi were maintained as pure cultures at Yunnan University (Kunming, China). Genomic DNA was extracted from purified fungi using the CTAB method described by Zolan et al. [51]. This served as the PCR template, with the universal primers ITS4 (5′-TCCTCCGCCTTATTGATATGC-3′) and ITS5 (5′-GGAAGTAAAAGTCGTAACAAGG-3′) amplifying an approximately 600-bp fragment of the ITS region [52]. The PCR conditions were as follows: predenaturation at 94 °C for 4 min; denaturation at 94 °C for 1 min, annealing at 54 °C for 1 min, and extension at 72 °C for 1 min, followed by 35 cycles; and final extension at 72 °C for 10 min. DNA sequencing was performed at Sangon Biotech Co., Ltd. (Shanghai, China).

### 2.4. Fungal DNA Extraction Without Cultivation

The aforementioned cellophane samples bearing airborne fungal spores and leaf tissue specimens were stored at −80 °C. DNA sequencing was performed at Lc-Bio Technologies Co., Ltd. (Hangzhou, China), encompassing a total of 108 samples across three categories. Fungal DNA extraction employed the TIANamp Bacteria DNA Kit DP302-02 (TIANGEN, Beijing, China) following the kit protocol. PCR amplification of the ITS2 region utilised the ITS1FI2 primer (5′-GTGARTCATCGAATCTTTG-3′) and ITS2 primer (5′-TCCTCCGCTTATTGATATGC-3′) [53]. PCR conditions: 95 °C predenaturation for 30 s; 95 °C denaturation for 10 s, 52 °C annealing for 30 s, 72 °C extension for 45 s, repeated for 32 cycles; final extension at 72 °C for 10 min. Employing ultrapure water throughout the DNA extraction process, rather than sample solution, eliminates the possibility of false-positive PCR results serving as negative controls. PCR products were purified using AMPure XT beads (Beckman Coulter Genomics, Danvers, MA, USA) and quantified with Qubit (Invitrogen, Carlsbad, CA, USA). The purified PCR products were assessed using Agilent 2100 Bioanalyzer (Agilent, Santa Clara, CA, USA) and Illumina (Kapa Biosciences, Woburn, MA, USA) library quantification kits. Qualified library concentrations should be above 2 nM. Qualified sequencing libraries (with nonrepetitive index sequences) were gradient diluted, mixed in proportion to the required sequencing volume, denatured to single-stranded DNA with NaOH, and then sequenced. Sequencing was performed using a NovaSeq 6000 sequencer for 2 × 250 bp paired-end sequencing with the NovaSeq 6000 SP Reagent Kit (500 cycles).

### 2.5. Sequence Processing

For first-generation sequencing results obtained via the isolation and culture method, the sequences of the culture-acquired strains were adjusted for correction via SeqMan version 7.0.0 (DNAstar 5.0) and aligned for shear redundancy via BioEdit version 7.0 [54]. The ITS sequence data generated in this study were compared with the available sequence data of the type or representative isolates in GenBank via the BLAST (https://blast.ncbi.nlm.nih.gov/Blast.cgi?PROGRAM=blastn&PAGE_TYPE=BlastSearch&LINK_LOC=blasthome (accessed on 10 December 2024)) function, and the sequences were classified into operational taxonomic units (OTUs) at the unique level via Mothur version 1.45.3, ultimately constructing an OTU-level abundance table. For next-generation sequencing data obtained via culture-free methods, samples must be split based on barcode information, with adapter and barcode sequences removed. Data assembly follows: first, primer sequences and balanced base sequences are removed from RawData (cutadapt v1.9), and then each pair of paired-end reads is merged into a longer tag via overlap regions (FLASH v1.2.8). Subsequently, a window-based quality scan was performed on the sequencing reads, with the default window size set to 100 bp. Where the average quality score within a window falls below 20, the portion of the read from the window’s start position to its 3’ terminus is truncated (fqtrim). Finally, data filtering was performed, including removal of sequences shorter than 100 bp after trimming, removal of sequences with N content (uncertain ambiguous bases) exceeding 5% after trimming, and removal of chimeric sequences (Vsearch v2.3.4). Length filtering and denoising were performed using DADA2 via the command ‘qiime dada2 denoise-paired’. ASV feature sequences and ASV abundance tables were obtained, and singleton ASVs were removed. Species annotation was performed on the ASV sequence files using the RDP and Unite databases (version 2024), with a confidence threshold of >0.7. Species abundance at each taxonomic level across all samples was statistically quantified based on the ASV abundance matrix.

### 2.6. Statistical Analysis

Data analysis for this study was conducted using R v4.3.4 and SPSS v25.0. Visualisation and graphing were performed on the Wekemo Bioincloud platform (www.bioincloud.tech (accessed on 10 December 2024)) [55] and the Omicstudio platform [56]. The FUNGuild database was used to predict the functional guilds of the fungi in the three types of samples obtained via the two methods, and the present study conducted the analysis more conservatively, selecting only the results with the highest confidence rankings for inclusion in the analysis (i.e., “confidence ranking” was “highly probable” and “probable”) [57]. The VennDiagram package in R was employed to analyse the number of shared OTUs/ASVs across different sample types. The ggalluvial package in R was utilised to calculate the relative abundance of various taxonomic groups across seasons and generate a Sankey diagram. The vegan package in R was employed to compute Simpson’s index for analysing fungal community α-diversity. For culture-free data, normalisation (minimum sequence count) was applied to eliminate sequencing depth-induced errors. The data were tested for normality, and the means were compared via a nonparametric multivariate test (Kruskal-Wallis test) (Tukey’s HSD, Bonferroni correction adjusted *p* < 0.05). The vegan package in R was employed to compute a difference matrix based on Bray-Curtis distances, visualise differences in community structure between samples, and quantify these differences via the Adonis function for permutation multivariate analysis of variance (PERMANOVA). In addition to analysing differences between months and seasons, differences in the fungal communities associated with each phenological period of *A. adenophora* were also analysed. As a perennial herb, *A. adenophora* blooms once a year at our investigation site: from February to April, the budding and flowering stages occur; from May to June, the seed maturity stage occurs; from July to October, the growth stage occurs; and from November to January, the dormant stage occurs. Additionally, random forest analysis was conducted using the randomForest and ggplot2 packages to identify fungal taxa responsible for significant differences in fungal communities across seasons or phenological stages. Decreasing correlation analysis (DCA) and principal coordinate analysis (CCA) were performed using the vegan package to determine associations between microbial communities and environmental factors. The pheatmap package was employed to construct heatmaps illustrating fungal distribution across seasons, with clustering analysis performed using the Ward. D2 function to calculate Euclidean distances. The ggtern package analysed fungal community sharing levels among three sample types within the same season to explore potential transmission pathways of foliar pathogenic fungi, resulting in the construction of a ternary phase diagram.

## 3. Results

**Figure 1:**
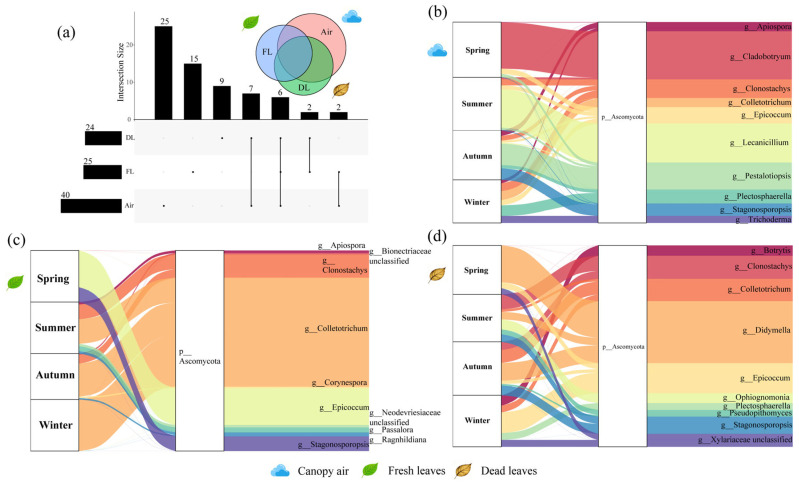
The composition of the pathogenic fungal communities associated with *A. adenophora*, (**a**) upset diagram of OTUs in the three sample types. Sankey diagrams of dominant genera and corresponding phyla with (**b**) canopy air, (**c**) fresh leaves and (**d**) dead leaves. Sankey diagrams show the distributions of the relative abundances of the top 10 dominant genera and corresponding phyla of culturable pathogenic fungi in different seasons. The “p_” and “g_” in the figure represent the phylum level and genus level in the classification level, respectively.

**Figure 2:**
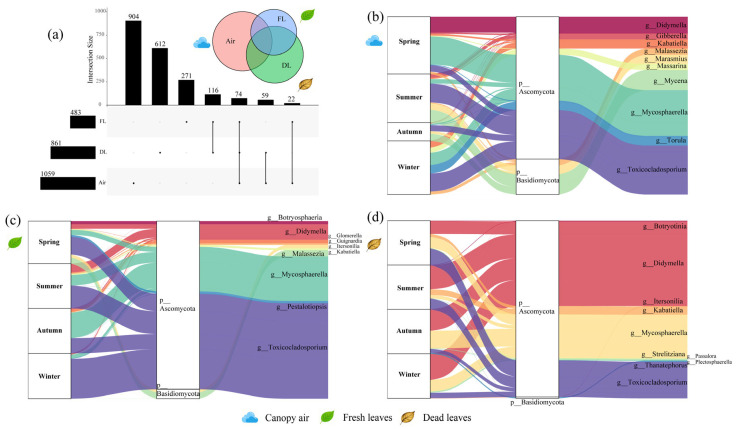
The composition of the pathogenic fungal communities associated with *A. adenophora*, (**a**) upset diagram of ASVs in the three sample types. Sankey diagrams of dominant genera and corresponding phyla with (**b**) canopy air, (**c**) fresh leaves and (**d**) dead leaves. Sankey diagrams show the distributions of the relative abundances of the top 10 dominant genera and corresponding phyla of unculturable pathogenic fungi in different seasons. The “p_” and “g_” in the figure represent the phylum level and genus level in the classification level, respectively.

A total of 9191 strains were obtained from ICM and categorised into 215 OTUs. A total of 119 OTUs were from air (40 of the pathotroph OTUs), 83 OTUs were from fresh leaves (25 of the pathotroph OTUs), and 66 OTUs were from the dead leaf community (24 of the pathotroph OTUs). A total of 9191 strains were identified across 68 known genera, encompassing 44 families and 3 phyla. DNA sequencing of 108 CFM samples yielded a total of 9,047,783 raw tags, with an average of 83,776 raw tags per sample. Following data filtering and species annotation database alignment, 8,806,853 Valid_Tags were confirmed as fungal sequences, averaging 81,545 Valid_Tags per sample. Fungi-specific sequences constituted 97.29% of the total valid sequences across all samples. The Goods coverage index for each sample’s dilution curve reached 100%, indicating that all species within the samples were sequenced. The filtered sequences thus fully represent fungal diversity (Figure S1). These Valid_Tags were classified into 8402 ASVs, of which 5701 ASVs were from air (1059 pathotroph ASVs), 1448 ASVs were from fresh leaf tissues (483 pathotroph ASVs), and 2220 ASVs were from dead leaf tissues (861 pathotroph ASVs) (Figure 1, Figure 2, Figures S2 and S3). These sequences were identified as belonging to 676 known genera, encompassing 281 families and 7 phyla. ICM results revealed that the air, fresh leaves, and dead leaves communities shared 13 OTUs, with 91, 53, and 29 unique OTUs, respectively. CFM results indicated that the three communities shared 182 ASVs, with 5255, 851, and 1511 unique ASVs, respectively. Both methods showed that the air harboured the highest number of unique fungi.

### 3.1. Predicted Trophic Mode of Fungi Associated with A. adenophora

**Figure 3:**
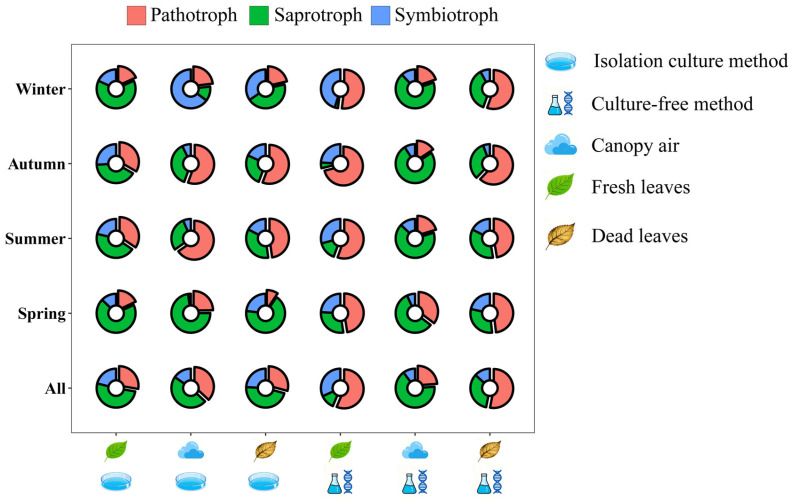
Double-layered circular plots of the relative abundance of fungi of different trophic modes in the three types of samples at the overall level and across seasons. The specific values are shown in Table S2.

The fungal trophic modes predicted from the FUNGuild database are shown in Figure 1 and Table S2; in this study, the results with high confidence were selected. At the annual level, ICM revealed that both the canopy air and leaf tissue fungal communities of *A. adenophora* were dominated by saprotrophs (46.57–51.09%). In contrast, CFM revealed that the fungal community associated with *A. adenophora* leaf tissue was dominated by pathotrophs (53.06–56.30%), whereas that associated with canopy air was dominated by saprotrophs (66.03%) (Figure 3, Table S2). In addition, there were variations in the percentage of fungi in each trophic mode across seasons and sites. ICM revealed that the number of pathotrophs was highest in summer or autumn among all three sample types, whereas the number of saprotrophs was highest in spring. Symbiotrophs, on the other hand, had the highest percentage in autumn or winter. The CFM results revealed that the seasonal patterns of pathotrophs were different in leaf tissues and air, with the highest prevalence in leaf tissues, mainly in autumn, and the highest prevalence in air in spring, whereas saprotrophs and symbiotrophs did not show significant seasonal patterns. Overall, pathotrophs presented the most pronounced seasonal patterns, and the patterns of change in leaf tissues were similar; therefore, pathotrophs were analysed separately in the results presented in the next section.

### 3.2. Fungal Community Structure Associated with A. adenophora

Both methods revealed that Ascomycota was the most dominant phylum in all the samples, but the results differed significantly at the genus level (Figures S2 and S3). In the air sample, the dominant genera obtained by ICM included *Cladosporium* (annual average relative abundance 61.52%), *Fusarium* (9.51%), *Alternaria* (4.84%), Didymellaceae unclassified (4.06%) and *Penicillium* (2.62%), whereas the CFM included *Davidiella* (25.49%), Fungi-unclassified (6.67%), *Mycosphaerella* (4.97%), *Hyphodontia* (4.01%) and *Psathyrella* (3.72%) (Figures S2b and S3b). In the fresh leaf sample, the dominant genera obtained by ICM included *Nemania* (20.19%), *Xylaria* (14.01%), *Colletotrichum* (11.28%), Didymellaceae unclassified (8.27%) and *Cladosporium* (7.31%), whereas the CFM included *Toxicocladosporium* (42.90%), *Mycosphaerella* (20.05%), *Didymella* (6.22%), Fungi_unclassified (3.62%) and *Davidiella* (3.07%) (Figures S2c and S3c). In the dead leaf sample, the dominant genera obtained by ICM included Didymellaceae unclassified (42.95%), *Xylaria* (11.00%), *Alternaria* (8.13%), *Cladosporium* (5.33%) and *Nemania* (4.96%), whereas the CFM included *Didymella* (38.63%), *Mycosphaerella* (20.11%), *Toxicocladosporium* (15.67%), *Davidiella* (6.72%) and *Kabatiella* (3.54%) (Figures S2d and S3d). Overall, the fungal taxa detected by the two methods differed significantly at the genus level, suggesting differences in the sensitivity of the two methods for isolating different taxa of fungi (culturable and uncultured).

The dominant culturable pathogenic fungi in the air samples included *Cladobotryum* (21.68%), *Lecanicillium* (17.67%), and *Pestalotiopsis* (12.09%), and the relative abundances of these genera varied considerably from season to season. The results revealed that *Cladobotryum* was most abundant in the spring, whereas in the summer, it became *Lecanicillium*, and in the autumn, it was *Pestalotiopsis*. In winter, the composition of the dominant populations became more diverse (Figure 1b). The dominant culturable pathogenic fungi in the fresh leaf samples included *Colletotrichum* (54.97%), *Epicoccum* (20.00%), and *Clonostachys* (6.27%), with *Epicoccum* being most dominant in the spring and *Colletotrichum* being most dominant in the other three seasons. Unlike those in air, the dominant populations in fresh leaves were more diverse in composition in summer (Figure 1c). The dominant culturable pathogenic fungi in the dead leaf samples included *Didymella* (28.16%), *Epicoccum* (13.62%) and *Clonostachys* (10.94%). The composition of the dominant populations in dead leaves was more diverse across seasons and did not show significant seasonal differences (Figure 1d). The dominant uncultured pathogenic fungi in all three types of samples were *Toxicocladosporium* (18.42–52.12%), *Mycosphaerella* (17.23–24.35%) and *Didymella* (6.35–47.04%), with the exception that the level of dominance varied from sample to sample (Figure 2). *Toxicocladosporium* (18.42%/52.12%) was most abundant in air and fresh leaves, whereas *Didymella* (47.04%) was most abundant in dead leaves (Figure 2c,d). Overall, the composition and abundance of the dominant populations of culturable pathogenic fungi mainly varied across seasons and sample types, whereas the compositions of the dominant communities of the uncultured pathogenic fungal communities were relatively stable, with only the abundance changing across seasons and sample types.

### 3.3. Diversity of Fungal Communities Associated with A. adenophora

**Figure 4:**
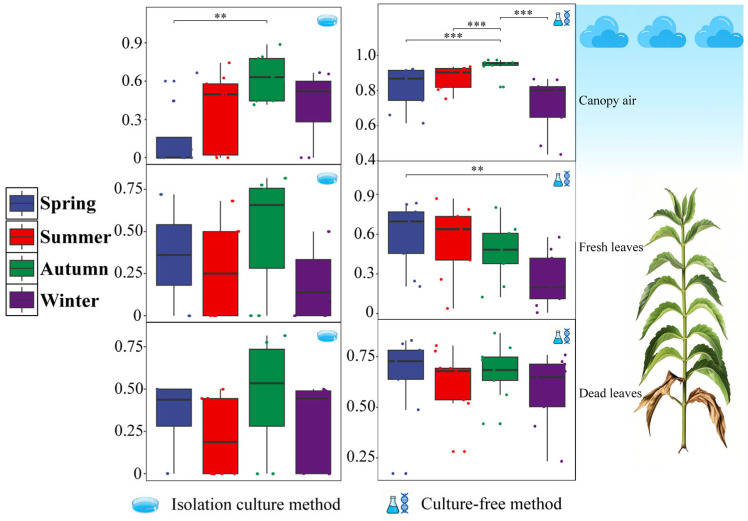
Simpson’s index of pathogenic fungal communities for each season for the three samples. The statistics show the mean and between-group differences at the seasonal level for each type of sample. The points in the box plot represent the actual values of each repetition within this data set. The colouring of the points corresponds to the colour of the box plot for the season to which they belong. Differences between groups were analysed via the Wilcoxon test, with the markers “**”, and “***” representing a significant difference (*p* < 0.01) and a highly significant difference (*p* < 0.001), respectively.

The diversity of the samples was assessed via Simpson’s index. The results revealed that the diversity of both types of fungal communities in air and fresh leaf tissues was highest in autumn and lowest in spring. The diversity of both types of fungal communities in dead leaf tissue did not change significantly with seasonal changes (Figure S4). When focusing only on pathogenic fungi, the results revealed that the diversity of both types of fungal communities in the air likewise reached its highest level in autumn. The diversity of culturable pathogenic fungi in fresh leaf tissue was highest in autumn, but the difference was not significant, while the uncultured pathogenic fungal community was highest in spring. Similarly, the diversity of the two types of fungal communities in dead leaf tissues was not significantly different from that in any of the other seasons, although the mean values were highest in autumn or spring (Figure 4). Overall, both types of fungal communities presented relatively consistent seasonal dynamics in terms of diversity, and seasonal changes significantly affected community diversity in air and fresh leaf tissues, except for culturable pathogenic fungi in fresh leaf tissues. In contrast, seasonal variation had the least effect on the diversity of both fungal communities in dead leaf tissue, with no significant differences between seasons.

PCoA further revealed differences in community structure across seasonal samples, while random forest analysis identified biological markers driving these variations. Overall community results (Figures S5 and S6) indicated that the cultivable fungal community structure of all three sample types exhibited highly significant differences between seasons (*F* values ranging from 1.799 to 2.384; *p* values all 0.001). Seasonal variation exhibited the highest explanatory power for airborne community differences, with the first and second principal coordinate axes cumulatively explaining 46.26%. Fallen leaves followed, cumulatively explaining 37.1%. Seasonal variation demonstrated the lowest explanatory power for fresh leaf communities, accounting for only 22.3%. The OTU contributing most significantly to differences in the air community was OTU2 Fusarium, followed by OTU44 *Lecanicillium* and OTU57 *Pestalotiopsis*. Within the fresh leaf community, OTU43 *Rachicladosporium* contributed most significantly to the differences, followed by OTU11 *Nemania* and OTU2 *Fusarium*. Within the dead leaf community, OTU8 Didymellaceae unclassified contributed most significantly to the differences, followed by OTU4 Didymellaceae unclassified and OTU1 *Cladosporium*. The structure of the uncultivable fungal communities across the three sample types also exhibited significant differences between seasons (*F* values ranging from 1.89 to 2.956; *p* values ranging from 0.007 to 0.001). Seasonal variation explained the highest proportion of variance in the leaf litter community (52.6%), followed by the fresh leaf community, with a cumulative explanation of 44.17%. Seasonal variation explained the lowest proportion of variance in the airborne community, at only 32.87%. Within the air community, ASV29 *Hyphodontia* contributed most significantly to variation, followed by ASV1 *Didymella* and ASV19 *Davidiella*. Within the fresh leaf community, ASV2 *Davidiella* contributed most significantly to variation, followed by ASV197 *Paraconiothyrium* and ASV1168 *Arthrinium*. In the dead leaf community, ASV42 *Plectosphaerella* contributed most significantly to variation, followed by ASV20 *Didymella* and ASV8 *Alternaria*.

**Figure 5:**
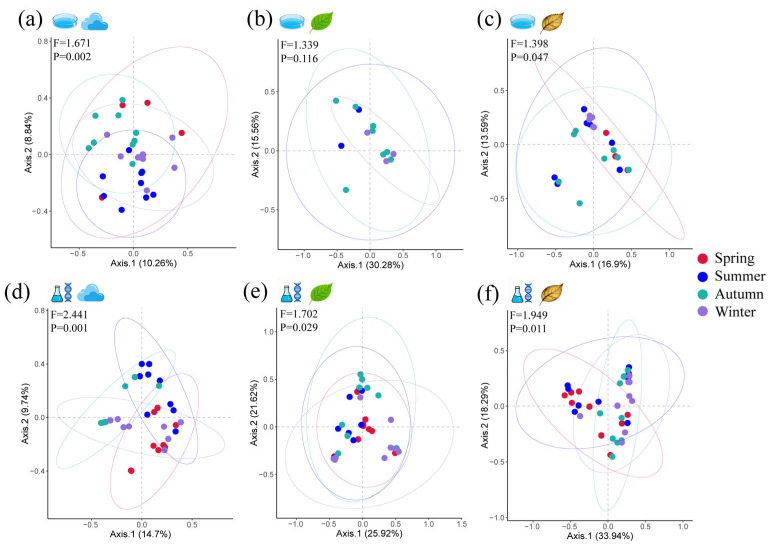
Principal coordinate analysis based on Bray–Curtis matrix distances of pathogenic fungal communities in (**a**,**d**) canopy air, (**b**,**e**) fresh leaves, and (**c**,**f**) dead leaves among different seasons. Matrix distances are calculated on the basis of (**a**–**c**) culturable and (**d**–**f**) unculturable fungi. Fewer culturable pathogenic fungi were obtained from fresh leaves in the spring, resulting in fewer than three analysable replicates, so they cannot be shown in (**b**). Differences between communities were quantified by PERMANOVA and are shown in the upper left corner of each subplot. The circles in the figure represent confidence ellipses at a 95% confidence level, indicating the distribution range of the sample points. The colours correspond to the colours of the seasonal sample points.

**Figure 6:**
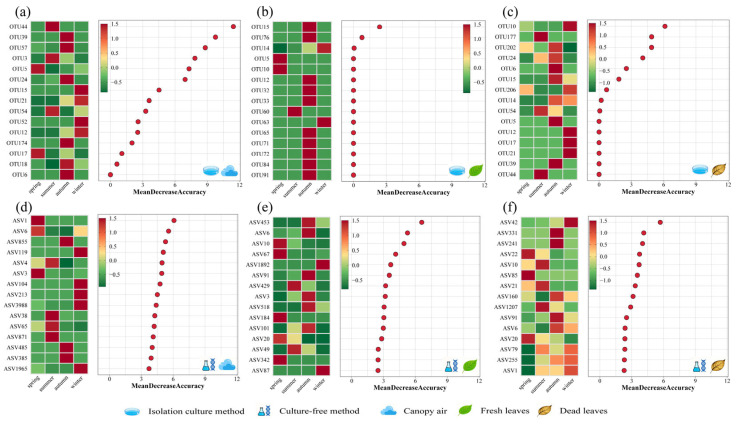
Random forest analysis of the pathogenic fungal communities in (**a**,**d**) canopy air, (**b**,**e**) fresh leaves, and (**c**,**f**) dead leaves in different seasons. Analysis was calculated on the basis of (**a**–**c**) culturable and (**d**–**f**) uncultured fungi. When the mean decrease accuracy value is greater than 0, it indicates that this OTU/ASV is a significant contributor to the seasonal differences in the pathogenic fungal community (*p* < 0.05).

When focusing solely on pathogenic fungi (Figure 5 and Figure 6), the fresh leaf community within the cultivable fungal group showed no significant seasonal variation (F = 1.339, *p* = 0.116). In contrast, both the airborne and litter communities exhibited significant differences (*F* values of 1.671 and 1.398, respectively; *p* values of 0.002 and 0.047, respectively). Seasonal variation explained 19.1% of the variance in airborne fungal communities and 30.49% of the variance in fallen leaf fungal communities. Within airborne communities, OTU44 *Lecanicillium* contributed most significantly to this variance, followed by OTU39 *Stagonosporopsis* and OTU57 *Pestalotiopsis*. Within the fresh leaf community, OTU15 *Rachicladosporium* contributed most significantly to variation, followed only by OTU76 *Colletotrichum*, whose contribution reached statistical significance. Within the fallen leaf community, OTU10 *Epicoccum* contributed most significantly to variation, followed by OTU177 *Ophiognomonia* and OTU202 *Didymella*. Regarding the uncultivable group, seasonal differences were significant across all three sample communities (*F* values ranging from 1.702 to 2.441; *p* values ranging from 0.029 to 0.001). Seasonal variation explained 24.44% of the variance in the air community, 47.54% in the fresh leaf community, and 52.23% in the dead leaf community. Within the airborne community, ASV1 *Didymella* contributed most significantly to variation, followed by ASV6 *Mycosphaerella* and ASV855 *Gymnopus*. Within the fresh leaf community, ASV453 *Mycosphaerella* contributed most significantly to variation, followed by ASV6 *Mycosphaerella* and ASV10 *Kabatiella*. In the dead leaf community, ASV42 *Plectosphaerella* contributed most significantly to variation, followed by ASV331 *Glomerella* and ASV241 *Mycosphaerella*. Overall, seasonal variation exerted no significant influence on cultivable pathogenic fungi within fresh leaf communities. It explained a higher proportion of variance in dry leaf communities but a lower proportion in fresh leaf communities. This indicates that the annual dynamics of fresh leaf communities are governed by nonseasonal factors to a greater extent.

**Figure 7:**
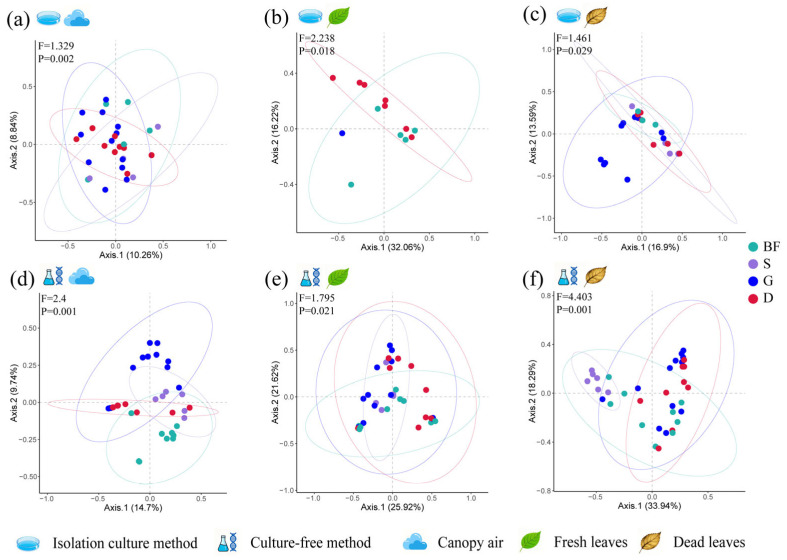
Principal coordinate analysis based on Bray–Curtis matrix distances of pathogenic fungal communities in (**a**,**d**) canopy air, (**b**,**e**) fresh leaves, and (**c**,**f**) dead leaves among different phenological periods. Matrix distances are calculated on the basis of (**a**–**c**) culturable and (**d**–**f**) uncultured fungi. Similarly, fewer culturable pathogens were obtained from fresh leaves during the growth period, resulting in fewer than three analysable replicates, which therefore cannot be shown in (**b**). Differences between communities were quantified by PERMANOVA and are shown in the upper left corner of each subplot. The circles in the figure represent confidence ellipses at a 95% confidence level, indicating the distribution range of the sample points. The colours correspond to the colours of the seasonal sample points.

In addition, we analysed the effects of the phenological period on fungal communities, including the budding and flowering stages, seed maturity stage, growth stage and dormant stage. The results from the analysis of the entire community revealed that the fungal community structure in all three samples was significantly influenced by the phenological stage of the glandular-hair fungus (*F* values ranging from 1.984 to 4.221; *p* values ranging from 0.004 to 0.001), with cumulative explanatory power between 24.44% and 52.6% (Figure S7). When focusing solely on pathogenic fungi, the results indicate that both types of pathogenic fungal communities were influenced by phenological stages, with all three samples exhibiting comparable degrees of influence (*F* values ranging from 1.461 to 4.403; *p* values ranging from 0.029 to 0.001). The cumulative explanatory power ranged between 22.3% and 52.6% (Figure 7). In contrast, the differences in pathogenic fungal communities in fresh and dead leaf tissues due to phenological period were smaller than the differences in overall community levels. Overall, the community structure of both types of fungi in air and leaf tissues was significantly affected by season and phenological period. With the exception of season, which had no effect on the community structure of culturable pathogenic fungi in fresh leaf tissues, the community structure of both types of pathogenic fungi in the remaining samples was also affected by season and phenological period but to a lesser extent in leaf tissues than at the overall community level.

### 3.4. Effects of Environmental Factors on Fungal Communities Associated with A. Adenophora

We further selected common factors with seasonal variations to analyse the effects of environmental factors on fungal communities, which included monthly mean temperature (T), monthly mean relative humidity (RH), monthly mean wind speed (WS) and total precipitation (TP). The results at the overall community level revealed that all four environmental factors affect the fungal community associated with *A. adenophora* to varying degrees, with the degree of effect depending on the type of fungal community and the environment in which it is located (Figure S8). For example, in air, culturable communities are affected only by temperature, whereas they are affected by all four factors in leaf tissue, where temperature is affected to a greater extent than in air. For uncultivated communities, all four factors were significantly affected in both air and leaf tissue but not by precipitation in fresh leaf tissue. Taken together, temperature had a greater effect on the overall community than the other environmental factors did, whereas precipitation had the least effect.

**Figure 8:**
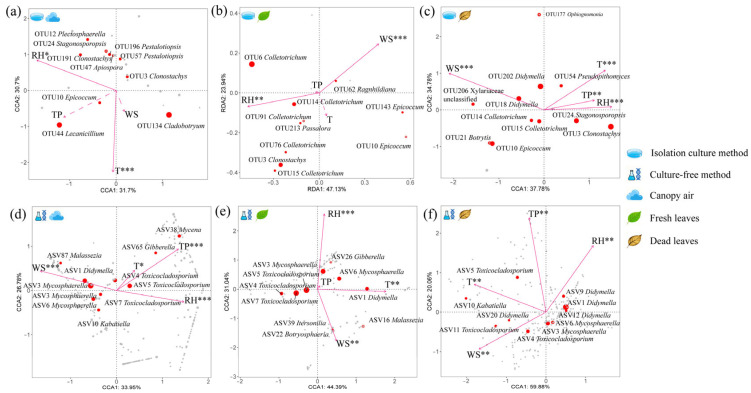
Canonical correspondence analysis or redundancy analysis of pathogenic fungal communities in (**a**,**d**) canopy air, (**b**,**e**) fresh leaves, and (**c**,**f**) dead leaves on the basis of decision curve analysis results. The patterns of response of (**a**–**c**)the top 10 OTUs and (**d**–**f**) the top 10 ASVs in terms of abundance to environmental factors are highlighted in the figure. All analyses were based on OTU/ASV levels. The environmental factor markers “*”, “**” and “***” represent effects (*p* < 0.05), significant effects (*p* < 0.01) and highly significant effects (*p* < 0.001), respectively. Dashed rays represent no effect. See Table S4 for specific values. The arrows in the figure represent different environmental factors, with longer arrows indicating a greater influence on the distribution of the studied organisms. Solid lines denote statistically significant correlations (*p* < 0.05), while dashed lines indicate non-significant correlations (*p* ≥ 0.05). The points in the figure represent OTUs or ASVs across the entire sample population, i.e., distinct fungal taxa. The top ten most abundant OTUs or ASVs are highlighted in red.

When focusing only on pathogenic fungal communities, these four environmental factors equally influenced the dynamics of both types of community structure (Figure 8). For example, in air, culturable communities are affected only by temperature and relative humidity (Figure 8a), and in fresh leaf tissue, they are affected only by relative humidity and wind speed (Figure 8b). In addition, unculturable communities are not affected by precipitation in fresh leaf tissue (Figure 8e). Taken together, relative humidity had a greater effect on pathogen communities than did other environmental factors, whereas precipitation had the least effect. Interestingly, there are differences in the effects of environmental factors on some of the same pathogenic fungi in different environments. Among the dominant OTUs of culturable pathogenic fungi, as exemplified by relative humidity, the abundance of OTU10 *Epicoccum* in air was positively correlated with relative humidity but negatively correlated with leaf tissues. The abundance of OTU3 *Clonostachys* in air was negatively correlated with relative humidity but positively correlated with leaf tissues (Figure 8a–c). The same is true for uncultured fungi, e.g., the abundance of ASV1 *Didymella* was positively correlated with relative humidity in air but negatively correlated with that in leaf tissue. The abundance of ASV3 *Mycosphaerella* was negatively correlated with relative humidity in both air and dead leaf tissue but positively correlated in fresh leaf tissue (Figure 8d–f). In summary, pathogenic communities are affected differently than the overall community is. Environmental factors also affect the same pathogenic fungi differently in different microenvironments.

### 3.5. Potential Modes of Transmission of Pathogenic Fungi Associated with Fresh Leaves of A. adenophora

In order to understand whether fungi associated with fresh leaf tissues are potentially transmitted through air or dead leaves, we further compared fungal sharing among three different sample types within the same season. When the fungal communities of fresh leaves and other sample types are shared within a given season, it is assumed that the fungi may have spread between fresh leaves and a given sample type (i.e., dead leaves or air) during that season. Therefore, our study can define two types of putative transmission pathways: the fresh leaf-air pathway (FAP), by which fungi spread directly from fresh leaves by producing spores, and the fresh leaf-dead leaf pathway (FDP), by which fungi are able to survive on dead leaves and subsequently spread through spores.

**Figure 9:**
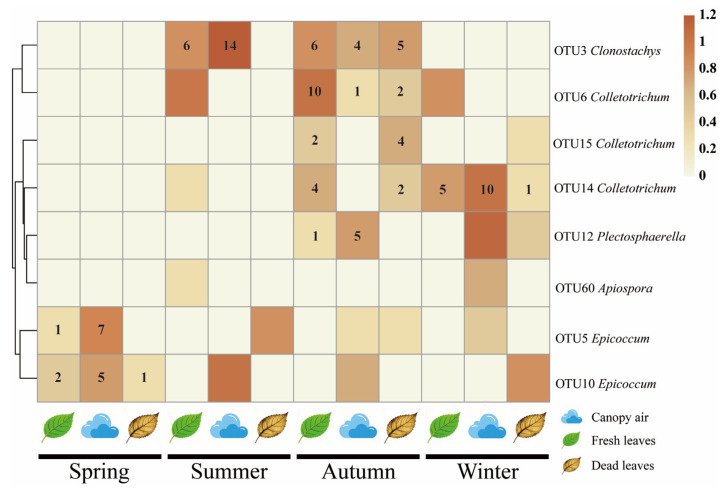
Heatmap of the distribution of culturable pathogenic fungi across the four seasons. Colouring is performed by taking the logarithm of the original values. The data in the heatmap represents the absolute abundance of the strains that are identified as potential transmission pathways. The figure indicates the absolute abundance values of pathogenic fungi with potential transmission phenomena during specific seasons.

Culturable pathogenic fungi of fresh leaf tissue were found to have potential transmission pathways throughout the year in a total of seven OTUs distributed across four genera, including *Clonostachys* (summer, FAP; autumn, FAP and FDP), *Colletotrichum* (autumn and winter, FAP and FDP), *Plectosphaerella* (autumn only, FAP) and *Epicoccum* (spring only, FAP and FDP) (Figure 9).

**Figure 10:**
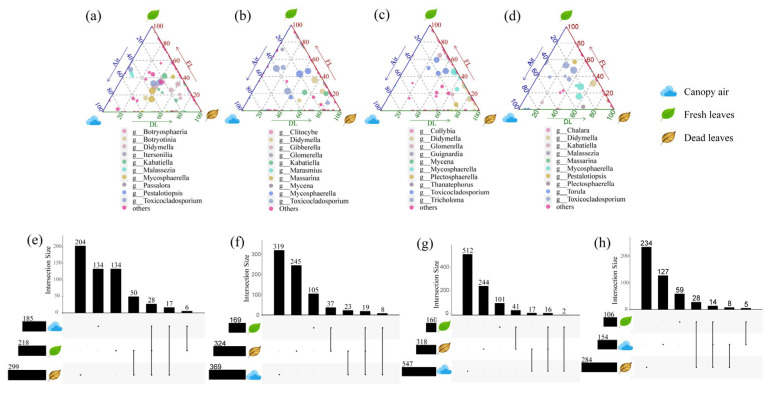
Ternary phase diagrams of the distribution of uncultured pathogenic fungi between the four seasons and upset plots of the sharing of ASVs. From left to right, the distribution and sharing correspond to (**a**,**e**) spring, (**b**,**f**) summer, (**c**,**g**) autumn and (**d**,**h**) winter.

A total of 146 ASVs with potential transmission routes were identified throughout the year in uncultured pathogenic fungi from fresh leaf tissue, which were distributed in 51 genera (Figure 10). Transmission was most active in the spring, when 36 genera with potential transmission pathways were detected. Winter had the lowest number of fungi, with only 22 genera detected with potential transmission pathways. The three most dominant genera throughout the year identified in the previous results, namely, *Toxicocladosporium*, *Mycosphaerella* and *Didymella*, were found to have potential spread throughout all four seasons. In addition, *Kabatiella, Malassezia*, *Plectosphaerella*, *Glomerella* and *Guignardia* were also found to have potential spread in all seasons. Other genera, such as *Itersonilia* (spring, autumn, and winter), *Botryotinia* (spring and winter), and *Chalara* (spring), are found to potential spread in only part of the season. For the putative transmission pathway, 22 of the 51 genera can be transmitted via both putative pathways throughout the year or part of the season. Among the genera that could spread throughout the year, only the most dominant, *Toxicocladosporium*, *Mycosphaerella* and *Didymella*, could spread via both pathways in all four seasons. In addition, only four genera, *Botryotinia*, *Chalara*, *Torula* and *Ophiognomonia*, could spread via the dual pathway in all the seasons in which they are found, but they can only spread in 1–2 seasons. The other 15 genera could only spread by one pathway in a given season; e.g., *Kabatiella* and *Malassezia* could spread only by FDP in autumn, *Malassezia* could spread only by FAP in summer, and *Plectosphaerella* could spread only by FDP in autumn. *Glomerella* could spread only by FDP present in the summer and winter. Finally, only 29 genera were transmissible via only one pathway, and 18 of these genera were found only in one season. Most of the genera with a single pathway of transmission have only putative FDP. Interestingly, putative FDP was not affected by season and was found every month. However, the putative FAP completely disappeared in October to December, and even *Toxicocladosporium*, *Mycosphaerella* and *Didymella* spread only through the putative FDP during these three months (Figure S9). Therefore, season was an important factor influencing the spread of foliar pathogenic fungi, with only a small number of species whose potential spread was not affected by season. Putative transmission pathways were also related to pathogenic fungal species, whereas season tended to limit FDP more weakly than FAP.

## 4. Discussion

### 4.1. The Potential of the Healthy Leaves of the Invasive Plant A. adenophora as a Reservoir of Pathogenic Fungi

Our study characterised pathogenic fungal communities in the leaf tissue of the invasive plant *A. adenophora* via ICM and CFM. The dominant genera of culturable pathogenic fungi found in fresh leaf tissues of *A. adenophora* included *Colletotrichum*, *Epicoccum* and *Clonostachys*. *Colletotrichum* has been reported to be a pathogen of many plants and is widely distributed worldwide [58,59]. However, *Colletotrichum* from healthy leaves suggests that it can exist as a leaf endophyte of *A. adenophora*. A previous study revealed that *Colletotrichum* commonly occurs in the foliage of *A. adenophora* without causing any symptoms but has some positive effects on *A. adenophora* in response to biotic and abiotic stresses [60]. Similarly, *Epicoccum* is widely reported as a plant pathogen but acts as a leaf endophyte of *A. adenophora* [61,62]. *Clonostachys*, on the other hand, has been reported to be a biocontrol fungus and was also found to be present in healthy leaves of *A. adenophora* in previous studies [63,64]. In contrast, the dominant genera of uncultivated pathogenic fungi on leaves included *Toxicocladosporium*, *Mycosphaerella* and *Didymella*. *Toxicocladosporium* belongs to Cladosporiaceae, a genus that includes a wide variety of plant endophytes, plant pathogens, and saprophytic fungi on plant foliage and in aqueous environments [65,66,67]. This also marks the first discovery of *Toxicocladosporium* as an endophytic fungus in fresh leaves of *A. adenophora*. *Mycosphaerella* (Mycosphaerellaceae) and *Didymella* (Didymellaceae) are also worldwide pathogens of a wide range of cash crops [68,69,70]. The occurrence of *Mycosphaerella* and *Didymella* in the leaf-associated tissues of *A. adenophora* has also been reported previously [48,71]. Our results once again confirm the colonisation of multiple plant pathogenic fungi on healthy leaves of *A. adenophora*, supporting the hypothesis that invasive plants may serve as reservoirs for pathogens.

### 4.2. Seasonal Dynamics of Pathogenic Fungal Community Composition and Diversity

We focused on seasonal changes in the composition and diversity of fungal pathogenic communities associated with *A. adenophora*. In the spring, the culturable pathogenic community was dominated by *Epicoccum* in the fresh leaves, whereas from summer to winter, it shifted to a dominance of *Colletotrichum*. The species composition of dead leaves was dominated by genera, including *Didymella* (most dominant in spring and autumn), *Epicoccum* (most dominant in winter) and *Clonostachys* (most dominant in summer). The results for unculturable taxa revealed that *Mycosphaerella* was most dominant in fresh leaf tissue in autumn, whereas *Toxicocladosporium* was most dominant in other seasons. In dead leaf tissue, *Toxicocladosporium* was most dominant only in spring, whereas *Didymella* was most dominant in the other seasons. Similar surveys in European forests have shown that the foliar fungal endophyte community composition of oaks and pines changes strongly during the growing season [37,72]; multiple genera of the phyllosphere fungal community of *Quercus macrocarpa* in the United States also show different seasonal patterns [73]. Random forest analysis indicates that *Epicoccum* is one of the greatest contributors to inter-seasonal variation within the pathogen community. As a widely distributed plant pathogen, *Epicoccum* has been reported throughout all seasons of the year [74]. This is linked to its multiple life strategies: when adopting a pathogenic strategy, it actively attacks and infects hosts, producing necrotic lesions [75]. This requires high humidity to facilitate transmission and infection; hence, disease reports are concentrated in tropical and subtropical regions from March to August. When environmental conditions are unfavourable for pathogenic transmission, *Epicoccum* shifts to a saprophytic lifestyle, persisting as endophytes within plant leaves or as saprophytes in necrophytic material via spores or ecological niche competition [76].

The diversity of culturable communities in fresh leaves of *A. adenophora* was highest in autumn; in contrast, the uncultivated community reached its highest level in spring. Previous studies on the seasonal dynamics of plant foliar fungal communities revealed that the overall abundance of foliar fungi in woody plants increased with the growing season to a maximum in autumn [37,72,73]. Studies on a wide range of cruciferous plants and herbaceous plants in grasslands have shown that fungal endophytes have the highest rate of colonisation in summer or autumn [77,78]. The seasonal dynamics exhibited by foliar endophytic fungi are now generally recognised as being due mainly to seasonal weather variations and changes in the physicochemical properties of leaves during the growing season of plants [37,73,79]. In our study, plants were sampled every month of the year, and fresh green leaves of *A. adenophora* were collected during the winter months. Therefore, it allows us to compare the effects of seasonal factors and phenological periods on changes in the foliar endophytic fungal community. We observed that the phenological period explained more community variability (*F* values in the range of 1.984–4.221) than did the seasonal factors (*F* values in the range of 1.799–2.956). The dynamic influence of phenological stages on phyllosphere microbial communities is primarily reflected in changes occurring within plant leaves during growth phases. Research indicates that the diversity and composition of endophytic microbial communities within olive phyllosphere are influenced by growth stages, independent of crop variety [80]. Existing studies suggest that variations in leaf phosphorus content during growth may constitute a key driver of phyllosphere microbial dynamics [81]. Furthermore, alterations in leaf physical structure accompanying changes in leaf age exert a significant impact on the colonisation and survival of phyllosphere microbes [82]. Consequently, phenological stages may offer a higher explanatory power for the dynamics of phyllosphere microorganisms.

For airborne communities, most studies suggest that airborne fungal diversity increases with increasing temperature [83,84]. We also observed the same results, with the highest diversity of airborne fungal communities occurring during autumn. Current research has widely confirmed that temperature is a key factor influencing fungal diversity in the air, with microclimates such as the urban heat island effect exerting a direct shaping influence on fungal communities [85,86]. Microclimates typically exert direct control over the physical dispersal of fungal spores and affect fungal germination, colonisation, and growth rates on host leaf surfaces [87]. Global climate change, however, represents a longer-term and more indirect driver of fungal thermal adaptation and pathogenicity shifts. Warming conditions may select for and enhance fungal heat tolerance, with certain pathogenic fungi exhibiting increased virulence adaptation under global warming scenarios [88,89]. When investigating numerous key processes affecting airborne fungal diversity at small scales (such as spore release), global climate models often fail to accurately characterise these mesoscale processes. However, microclimate and global climate change interact reciprocally. The manifestation of fungal diversity within microclimates may provide valuable reference for future studies examining fungal adaptive dynamics within a global climate context [90]. Unlike studies on foliar endophytes and airborne fungal communities, relatively few studies have been conducted on dead leaves. The dead leaves observed in this study included only those that had naturally turned yellow and had not yet fallen off the stem. Our results indicate that the diversity of fungal communities in dead leaves was not affected by seasonal changes, but community structure differed significantly between seasons. These findings suggest that seasonal changes in the fungal community associated with dead leaves do not add new species but rather species turnover [91]. Predictably, leaf senescence involves changes in leaf physicochemical factors and damage to the leaf cuticle. Leaves lose their selective filtering ability for fungi, which leads to a reduction in diversity between seasons [37,91]. Notably, we found that the dominant culturable pathogenic fungi in the air samples, including *Cladobotryum*, *Lecanicillium* and *Pestalotiopsis,* varied considerably from season to season. These genera are rarely reported in the literature, suggesting these fungi may be unique air-borne species in the vegetation invaded by *A. adenophora*, with unknown ecological functions.

### 4.3. Environmental Factors Accounting for Community Dynamics

Four common environmental factors, including temperature, relative humidity, wind speed and precipitation, were selected for analysis of factors explaining the community dynamics. The effects of temperature and relative humidity were stronger than those of wind speed and precipitation, with temperature having the greatest effect on fungal communities. Indeed, temperature and relative humidity have now been identified as important indicators for driving changes in air- and plant-associated fungal communities [17,83,92]. Moreover, relative humidity, temperature and their interactions are the most important indicators of air fungal spore density on all time scales. For example, airborne fungal spore abundance is consistently higher when the air is cool and moist [93]. Temperature and relative humidity are also equally important factors affecting phyllosphere microbial communities [18,94]. In general, either too high or too low temperatures can negatively affect foliar fungal communities, and community diversity tends to peak in spring or autumn [37,73,95]. The climate of the area where our sample site is located is characterised by high temperatures and rain in summer but cold and dry conditions in winter. Therefore, the results of our survey also revealed that the peaks in the diversity of fungal communities associated with *A. adenophora* occurred mostly in spring and autumn.

Interestingly, when we focused on the pathogenic fungal community, relative humidity was the strongest factor. Many studies have shown that high relative humidity is an important factor in the emergence and development of several fungal diseases [96,97]. When there is sufficient moisture, pathogenic fungi use water as a vector to increase the chances of successful attack on leaves [98]; pathogenic fungi can also benefit from high relative humidity (RH% > 90%) conditions to create an aqueous environment in the intercellular space, which, when extended to macroscopic visibility, is called water-soaked lesions [36]. Therefore, it is feasible and important to predict and control plant diseases by monitoring changes in relative humidity. Nonetheless, the effect of temperature on pathogenic fungi should not be ignored. Temperature strongly affects the efficiency of infestation and colonisation by primary pathogens. Unlike other trophic modes of fungi, pathotrophs appear to be more tolerant of temperature changes, and high temperatures increase the ability of pathogenic fungi to produce appressoria to invade plants or release more virulence factors to exacerbate infections [39,99,100]. It has also been shown that increased temperatures have more negative effects on pathogenic fungi in the phyllosphere [37,101].

Notably, relative humidity does not always have a positive effect on pathogenic fungal populations. We observed different effects of relative humidity on the same species in different microenvironments. For example, for the dominant species of the culturable community, relative humidity negatively affected OTU3 *Clonostachys* in the air but positively affected it in the leaf tissue. A similar situation was observed for ASV1 *Didymella* in uncultured communities. These results suggest that certain species respond to RH in different ways in different environments. We assumed that this is related to differences in the morphology of the fungus in different environments. For example, studies have shown that fungi are present mostly in plant tissues in the form of mycelia, which help them infest cells and obtain nutrients [102,103]. However, fungi usually exist as spores in the air to allow them to survive environmental stresses better for long-distance transmission. Rainfall promotes the release of fungal spores into the air, suggesting that high humidity is important for spore survival and transport [72,104]. The effect of high humidity on the spread and infestation of pathogens in plant tissues is also always positive, but most of these studies have focused on soil-borne bacterial pathogens [24]. Our results point to a potentially more complex scenario for the response of fungal pathogens to humidity; thus, it is necessary to further explore whether existing mechanisms can explain how environmental factors drive airborne fungal diseases. The rainy season in our sample area was mainly concentrated in summer and autumn, and in the results of the survey, we also found that the diversity of prototypical fungi of *A. adenophora* foliar pathogens peaked in autumn (culturable communities) or spring (uncultivated communities). Predictably, there are seasonal differences in the potential risk of disease spread from the invasion of *A. adenophora* in the study area. During seasons with increased precipitation frequency, high relative humidity for a short period of time after precipitation can lead to an increased abundance of pathogenic fungi [38]. For now, it is critical to focus on how a given pathogenic taxon of *A. adenophora* spreads during a given season.

### 4.4. Potential Transmission Patterns of Pathogenic Fungi

We further analysed two types of potential transmission pathways for pathogenic fungi in a given season: fresh leaf-air (FAP) and fresh leaf-dead leaf (FDP). We observed that 55 genera of both culturable and unculturable pathogenic fungi experienced various transmission pathways across seasons. Among them, transmission was detected in 16 genera in more than 3 seasons, and more than two-thirds (39/55) of the genera had detectable potential transmission in only 1–2 seasons. Culturable pathogenic fungi were more active in autumn, whereas uncultured pathogenic fungi were more prevalent in spring. As already mentioned, environmental conditions can strongly influence the release and spread of fungal spores, leading to differences in the level of transmission between seasons [105]. Plant disease occurrence is also seasonal [106]. Our data indicated that foliar pathogenic fungi were active in spring and autumn. There was a small difference between the mean spring and fall temperatures in the area where the study was conducted (spring/autumn: 17.8/16.1 °C), whereas there was a large difference in the mean relative humidity (spring/autumn: 42.74/76.73%). These findings suggest that pathogenic fungi have different evolutionary directions for humidity adaptation.

In addition, nine genera of pathogenic fungi were transmitted throughout all the seasons. We assumed that the seasonally unrestricted spread of these fungi is due to their wider host range and greater adaptation to environmental stresses. Pathogenic fungi with a larger range of susceptible hosts have greater ecological niche breadth, are more adaptable to their environment, and can maintain their transmissibility and pathogenicity over a wider range of temperature or relative humidity variations [107]. Accordingly, these nine highly transmissible pathogenic fungi indeed possess a wider pathogenic range as well as greater pathogenicity, e.g., *Toxicocladosporium* and *Mycosphaerella*. Studies have shown that *Toxicocladosporium* is globally widespread, has the ability to colonise different substrates and plants and has been found to be a pathogen of a wide variety of plants [66]. *Mycosphaerella* is one of the largest genera of ascomycetes, and the genus contains species that are found as pathogens on a wide range of plants, causing a wide range of plant diseases, including leaf spot [108].

With respect to transmission pathways, it is currently recognised that the transmission of pathogens involves two main pathways: horizontal transmission and vertical transmission. The mode of transmission is related to the adaptive evolution of the pathogen itself and is strongly influenced by environmental changes [109]. In addition, the nutritional mode of pathogens plays a nonnegligible role in the pathway of pathogen transmission. The biotrophic pathogen can only survive in living tissue and spreads directly from fresh leaves by producing spores with an incubation period [110]. In contrast, necrotrophic pathogens are able to survive on dead organisms because of their adaptive evolution into pathogenic and saprophytic pathogens [111], which enables them to survive the wilting of leaves and subsequently spread by spores [112]. This study focused on spore-based horizontal transmission, including the potential FAP and FDP. The results of the present study revealed that 27 of the 55 genera that dispersed were associated with the FAP and that 53 were associated with the FDP. These data suggest that most foliar fungal pathogens associated with *A. adenophora* are likely necrotrophic. Moreover, 25 of the 55 genera are capable of transmission via both pathways, suggesting a trade-off between different evolutionary modes and stability (i.e., evolutionary bi-stability) [109]. For example, *Mycosphaerella* has been reported to overwinter on fallen leaves until spring, when it spreads through spores. When no leaf litter is produced, the fungus can also spread by releasing spores directly on fresh leaves [73,113]. This evolutionary trait allows the pathogen to persist during seasons of high environmental stress or low plant biomass [114].

Notably, the uncultured pathogenic fungi on the foliage were not airborne during October to December, and we assumed that this phenomenon was related to the sudden decrease in precipitation. Precipitation in the study area decreases precipitously in late autumn, resulting in a reduction in the occurrence of short periods of high relative humidity, which severely affects the spread and infection levels of pathogenic fungi. These findings suggest that although these pathogenic fungi have evolved greater temperature and humidity tolerance, certain extreme environmental factors can still limit their spread.

## 5. Conclusions

This study investigated the seasonal changes in the fungal pathogenic community associated with the invasive plant *A. adenophora* over the course of a year, systematically analysed the environmental drivers, and identified their potential transmission modes. We have not performed an investigation to establish a direct relationship between disease occurrence in local plants and the transmission of fungal communities associated with *A. adenophora* in this case. However, our previous studies confirmed that *A. adenophora* foliage shares a variety of native plant leaf spot pathogens and experimentally verified the pathogenicity of most of these fungal species on native plants, mainly *Colletotrichum* and *Alternaria* [44]. Furthermore, our team verified that *A. adenophora* is capable of spreading *Colletotrichum* to native plants and crops both in vivo and in vitro, causing disease [115]. Therefore, it is highly possible that the high disease risk is driven by *A. adenophora* invasion in the local ecosystem. Future research will need to seek further evidence of the spread of these various pathogens discovered in *A. adenophora* to local plants and expand the research to more invasive plants to understand whether this potential transmission risk has commonalities.

## Data Availability

The fungal gene data obtained in this study have been uploaded to Genebank and Sequence Read Archive (SRA) of the National Center of Biotechnology Information. Gene nucleotide sequences obtained by isolation and culture method are available at GenBank: PV596793—PV597832. Fungal sequences obtained by culture-free method have been submitted and stored under Bioproject PRJNA1256322. Climate data used in the study were obtained from the China Meteorological Data Service Centre (https://data.cma.cn/ (accessed on 30 August 2023)).

## Supplementary Materials

The following supporting information can be downloaded at: https://www.mdpi.com/article/10.3390/microorganisms14010084/s1, Figure S1. Rarefaction curves using Good’s coverage estimator for fungal sequencing data from three types of samples associated with *A. adenophorum*. Air represents canopy air samples, FL represents fresh leaf samples, DL represents dead leaf samples, and the subsequent numbers indicate the month. Figure S2. The composition of the overall fungal communities associated with *A. adenophora*, (a) upset diagram of OTUs in the three sample types. Sankey diagrams of dominant genera and corresponding phyla with (b) canopy air, (c) fresh leaves and (d) dead leaves. Sankey diagrams show the distributions of the relative abundances of the top 10 dominant genera and corresponding phyla of culturable pathogenic fungi in different seasons. The “p_” and “g_” in the figure represent the phylum level and genus level in the classification level, respectively. Figure S3. The composition of the overall fungal communities associated with *A. adenophora*, (a) upset diagram of ASVs in the three sample types. Sankey diagrams of dominant genera and corresponding phyla with (b) canopy air, (c) fresh leaves and (d) dead leaves. Sankey diagrams show the distributions of the relative abundances of the top 10 dominant genera and corresponding phyla of unculturable pathogenic fungi in different seasons. The “p_” and “g_” in the figure represent the phylum level and genus level in the classification level, respectively. Figure S4. Simpson’s index of overall fungal communities for each season for the three samples. The statistics show the mean and between-group differences at the seasonal level for each type of sample. Differences between groups were analysed via the Wilcoxon test, with the markers “*”, “**”, and “***” representing a difference (*p* < 0.05), a significant difference (*p* < 0.01), and a highly significant difference (*p* < 0.001), respectively. Figure S5. Principal coordinate analysis based on Bray–Curtis matrix distances of overall fungal communities in (a,d) canopy air, (b,e) fresh leaves, and (c,f) dead leaves among different seasons. Matrix distances are calculated on the basis of (a–c) culturable and (d–f) unculturable fungi. Differences between communities were quantified by PERMANOVA and are shown in the upper left corner of each subplot. Figure S6. Random forest analysis of the overall fungal communities in (a,d) canopy air, (b,e) fresh leaves, and (c,f) dead leaves in different seasons. Analysis was calculated on the basis of (a–c) culturable and (d–f) uncultured fungi. When the mean decrease accuracy value is greater than 0, it indicates that this OTU/ASV is a significant contributor to the seasonal differences in the pathogenic fungal community (*p* < 0.05). Figure S7. Principal coordinate analysis based on Bray–Curtis matrix distances of overall fungal communities in (a,d) canopy air, (b,e) fresh leaves, and (c,f) dead leaves among different phenological periods. Matrix distances are calculated on the basis of (a–c) culturable fungi and (d–f) unculturable fungi. Differences between communities were quantified by PERMANOVA and are shown in the upper left corner of each subplot. Figure S8. Canonical correspondence analysis or redundancy analysis of overall fungal communities in (a,d) canopy air, (b,e) fresh leaves, and (c,f) dead leaves on the basis of decision curve analysis results. The patterns of response of (a–c) the top 10 OTUs and (d–f) the top 10 ASVs in terms of abundance to environmental factors are highlighted in the figure. All analyses were based on OTU/ASV levels. The environmental factor markers “*”, “**” and “***” represent effects (*p* < 0.05), significant effects (*p* < 0.01) and highly significant effects (*p* < 0.001), respectively. Dashed rays represent no effect. See Table S3 for specific values. Figure S9. (a) Venn diagram of the sharing of endophytic fungi in fresh leaf tissues of *A. adenophora* throughout the year and canopy airborne fungal communities from October to December at the ASV level. (b) Heatmap of the distribution of pathogenic fungi at the ASV level for the top 15 relative abundances of each of the above communities. Table S1. Sampling month and environmental factors. Table S2. Relative abundance of fungal trophic modes across seasons on the basis of the FUNGuild database. Table S3. Correlations between fungal community structure and environmental factors analysed via CCA. Table S4. Correlations between pathogenic fungal community structure and environmental factors analysed via CCA and RDA.
