# Supplementary material for: Seasonal Dynamics of Foliar Fungi Associated with the Invasive Plant Ageratina adenophora
Source: Microorganisms. 2025 Dec 30;14(1):84. doi: 10.3390/microorganisms14010084 (PMC12844525; doi:10.3390/microorganisms14010084)
Supplement: Supplementary file 1 [file microorganisms-14-00084-s001.zip › microorganisms-4020251-supplementary.pdf]

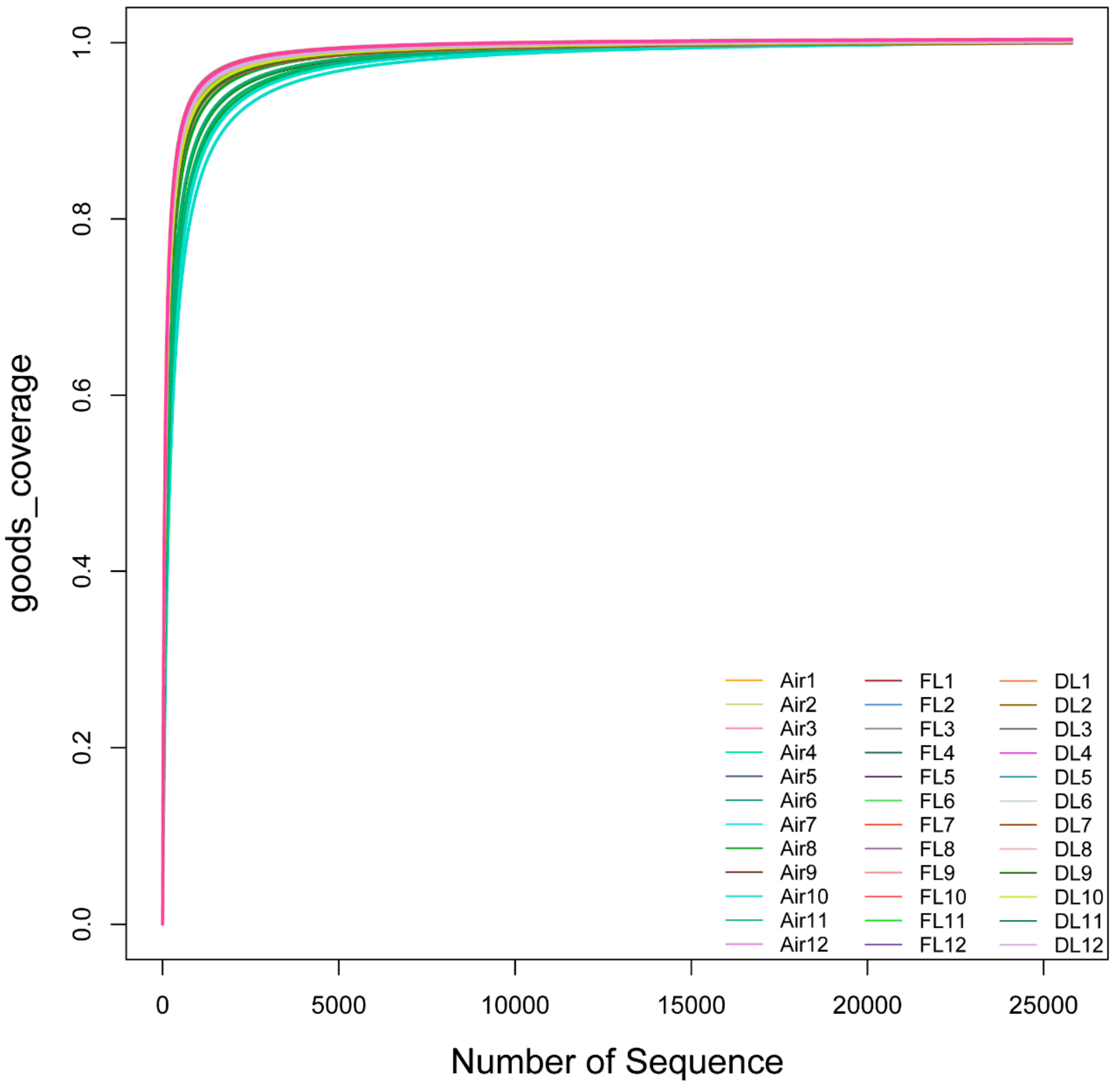

Figure S1. Rarefaction curves using Good's coverage estimator for fungal sequencing data from three types of samples associated with *A. adenophorum*. Air represents canopy air samples, FL represents fresh leaf samples, DL represents dead leaf samples, and the subsequent numbers indicate the month.

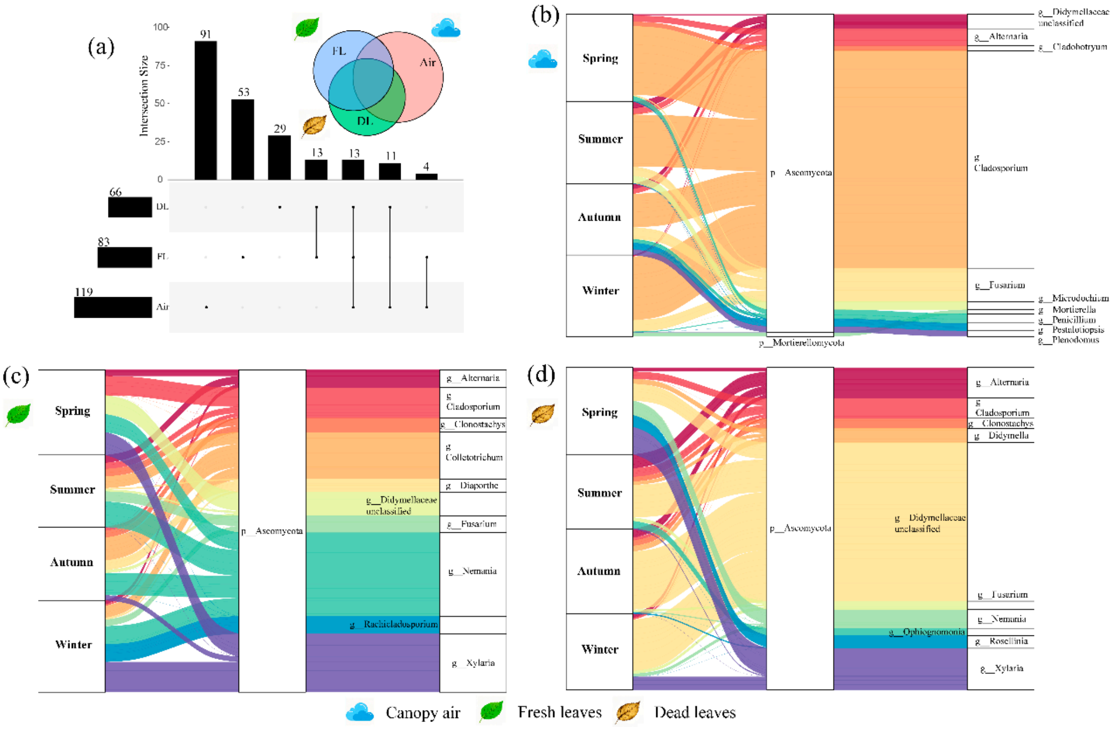

Figure S2. The composition of the overall fungal communities associated with *A. adenophora*, (**a**) upset diagram of OTUs in the three sample types. Sankey diagrams of dominant genera and corresponding phyla with (**b**) canopy air, (**c**) fresh leaves and (**d**) dead leaves. Sankey diagrams show the distributions of the relative abundances of the top 10 dominant genera and corresponding phyla of culturable pathogenic fungi in different seasons. The "p_" and "g_" in the figure represent the phylum level and genus level in the classification level, respectively.

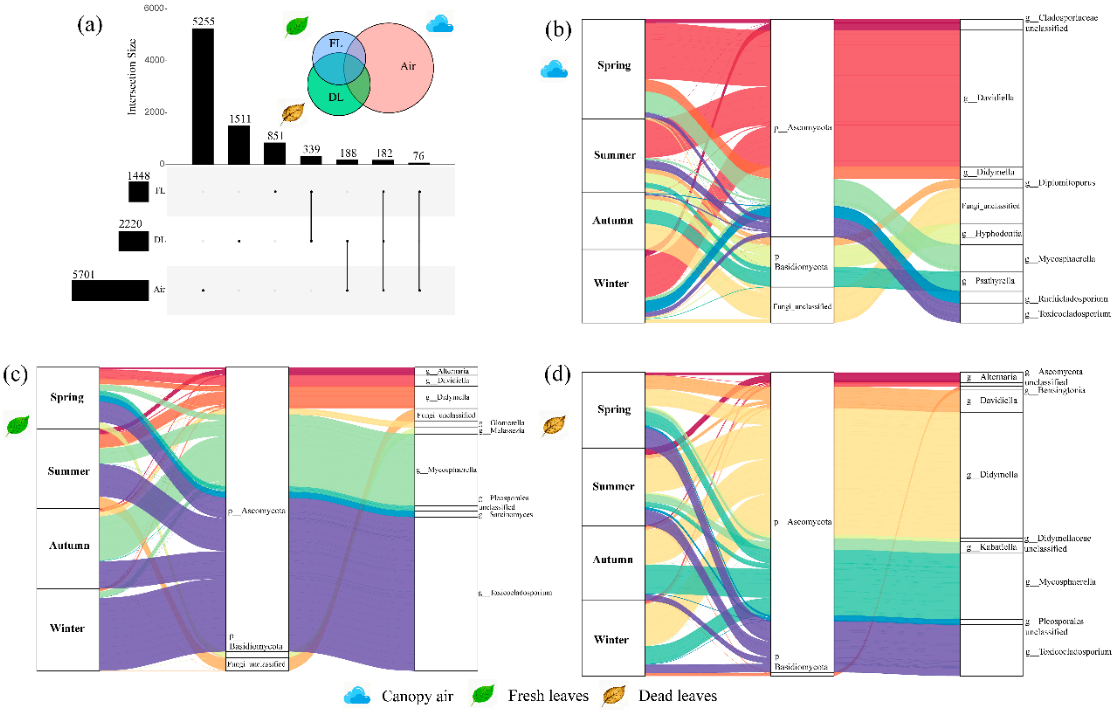

Figure S3. The composition of the overall fungal communities associated with *A. adenophora*, (**a**) upset diagram of ASVs in the three sample types. Sankey diagrams of dominant genera and corresponding phyla with (**b**) canopy air, (**c**) fresh leaves and (**d**) dead leaves. Sankey diagrams show the distributions of the relative abundances of the top 10 dominant genera and corresponding phyla of unculturable pathogenic fungi in different seasons. The "p_" and "g_" in the figure represent the phylum level and genus level in the classification level, respectively.

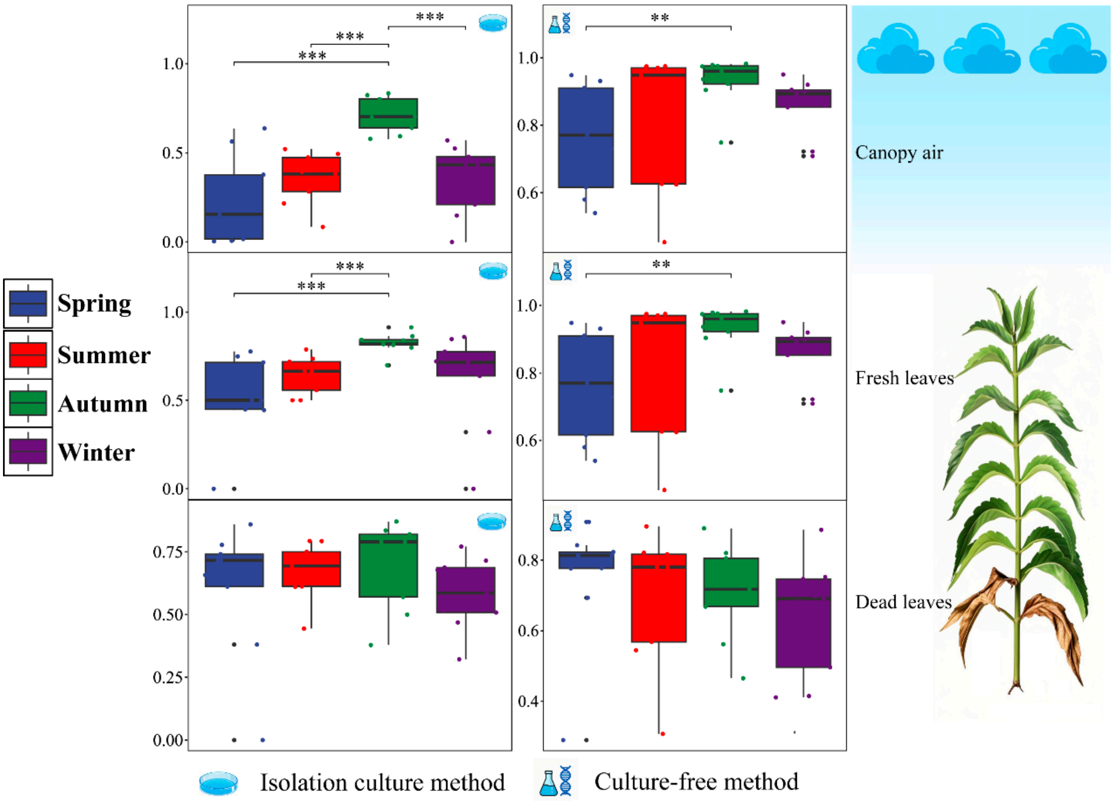

Figure S4. Simpson's index of overall fungal communities for each season for the three samples. The statistics show the mean and between-group differences at the seasonal level for each type of sample. Differences between groups were analysed via the Wilcoxon test, with the markers "*", "**", and "***" representing a difference (p<0.05), a significant difference (p<0.01), and a highly significant difference (p<0.001), respectively.

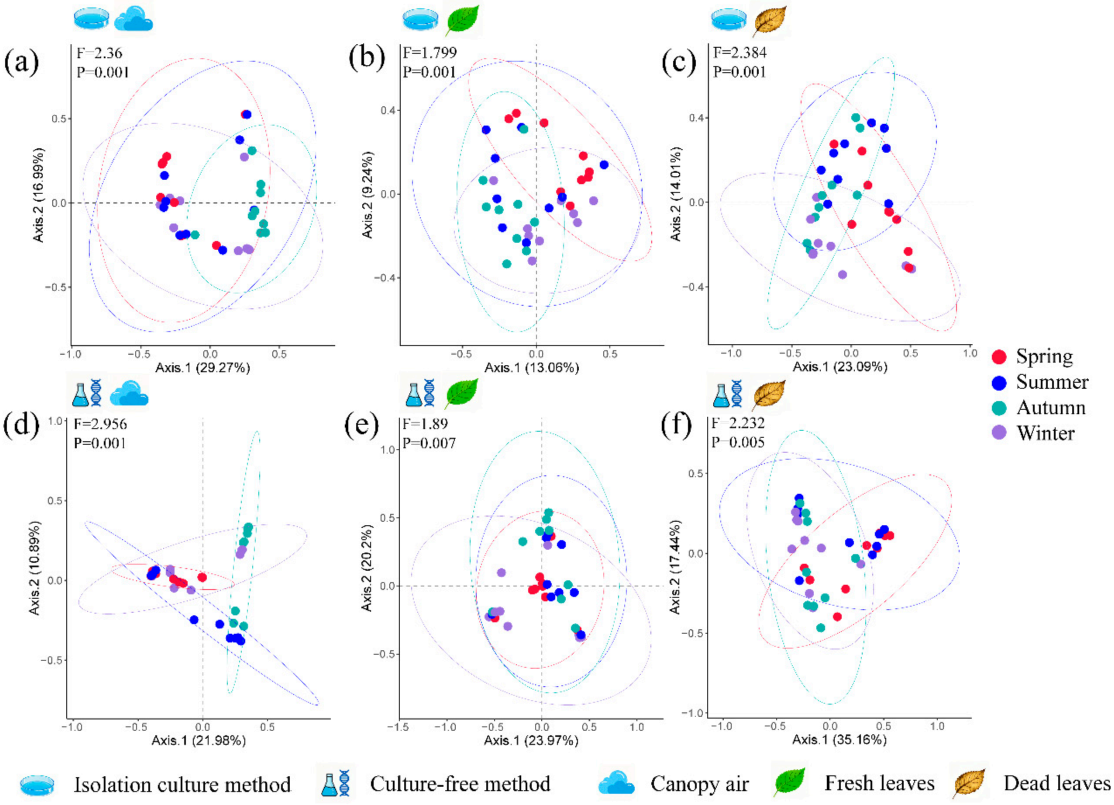

Figure S5. Principal coordinate analysis based on Bray-Curtis matrix distances of overall fungal communities in (**a**) (**d**) canopy air, (**b**) (**e**) fresh leaves, and (**c**) (**f**) dead leaves among different seasons. Matrix distances are calculated on the basis of (**a**) (**b**) (**c**) culturable and (**d**) (**e**) (**f**) unculturable fungi. Differences between communities were quantified by PERMANOVA and are shown in the upper left corner of each subplot.

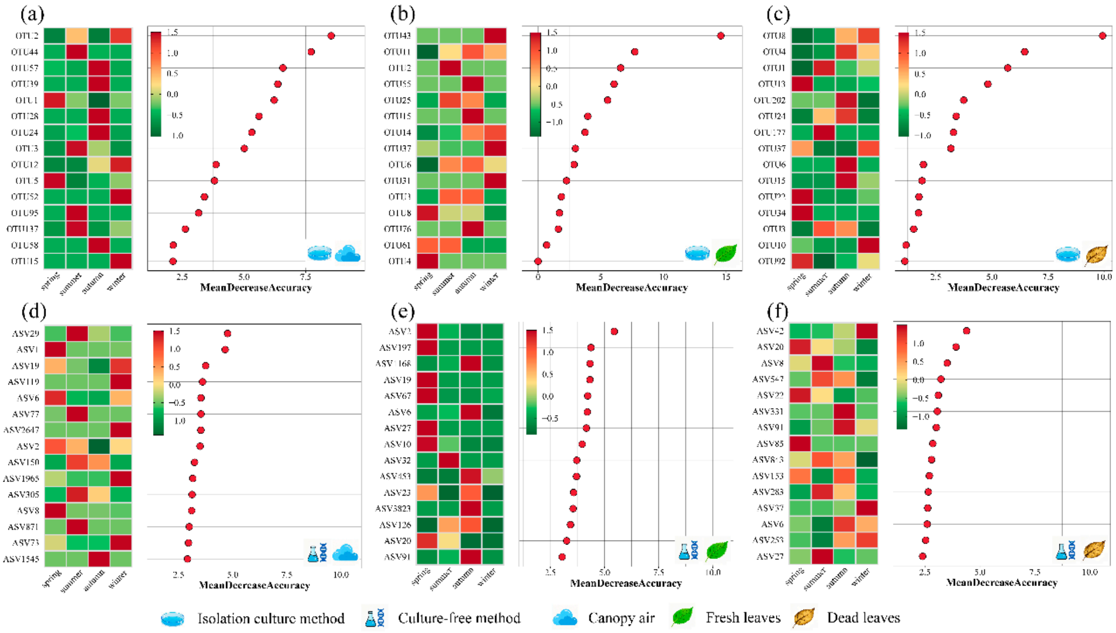

Figure S6. Random forest analysis of the overall fungal communities in (**a**) (**d**) canopy air, (**b**) (**e**) fresh leaves, and (**c**) (**f**) dead leaves in different seasons. Analysis are calculated on the basis of (**a**) (**b**) (**c**) culturable and (**d**) (**e**) (**f**) uncultured fungi. When the Mean Decrease Accuracy value is greater than 0, it indicates that this OTU/ASV is a significant contributor to the seasonal differences in the pathogenic fungal community ($p < 0.05$).

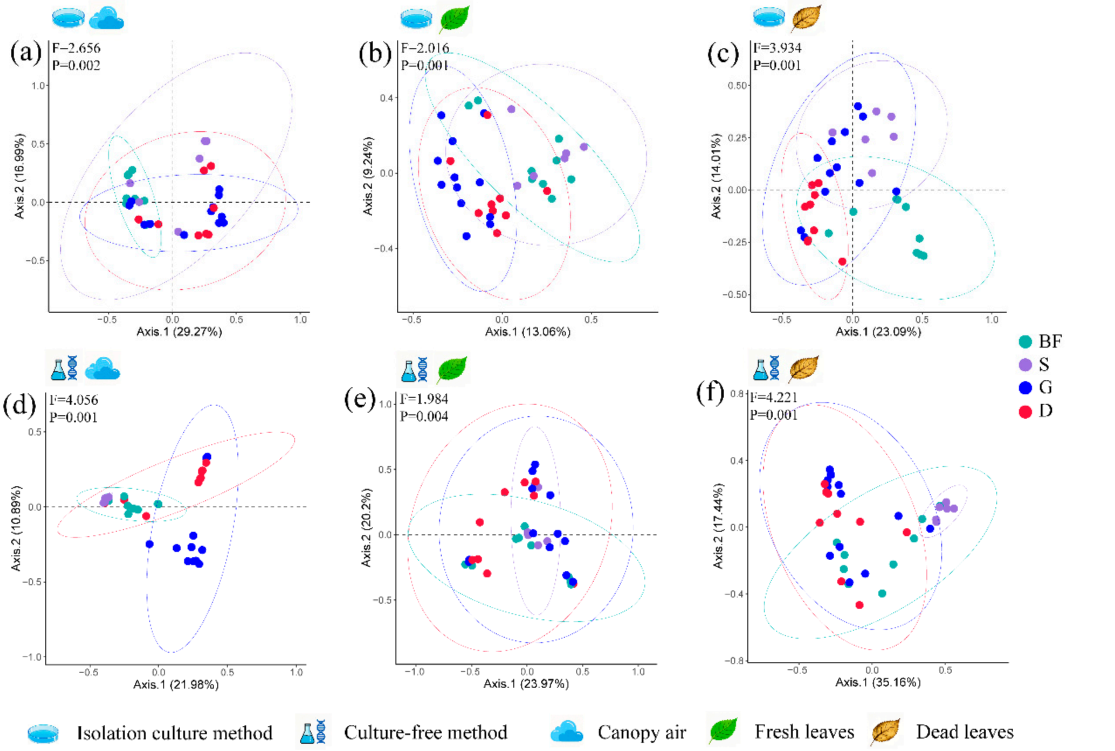

Figure S7. Principal coordinate analysis based on Bray-Curtis matrix distances of overall fungal communities in (**a**) (**d**) canopy air, (**b**) (**e**) fresh leaves, and (**c**) (**f**) dead leaves among different phenological

periods. Matrix distances are calculated on the basis of (**a**) (**b**) (**c**) culturable fungi and (**d**) (**e**) (**f**) unculturable fungi. Differences between communities were quantified by PERMANOVA and are shown in the upper left corner of each subplot.

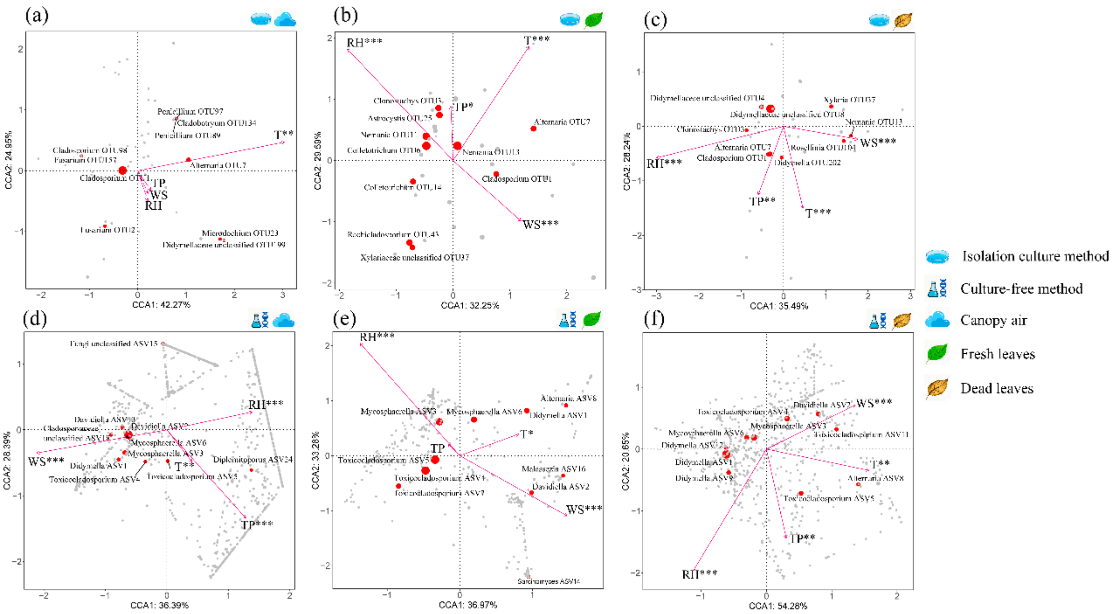

Figure S8. Canonical correspondence analysis or redundancy analysis of overall fungal communities in (**a**) (**d**) canopy air, (**b**) (**e**) fresh leaves, and (**c**) (**f**) dead leaves on the basis of decision curve analysis results. The patterns of response of (**a**) (**b**) (**c**)the top 10 OTUs and (**d**) (**e**) (**f**) the top 10 ASVs in terms of abundance to environmental factors are highlighted in the figure. All analyses were based on OTU/ASV levels. The environmental factor markers "*", "**" and "***" represent effects (P<0.05), significant effects (P<0.01) and highly significant effects (P< 0.001), respectively. Dashed rays represent no effect. See Table s3 for specific values.

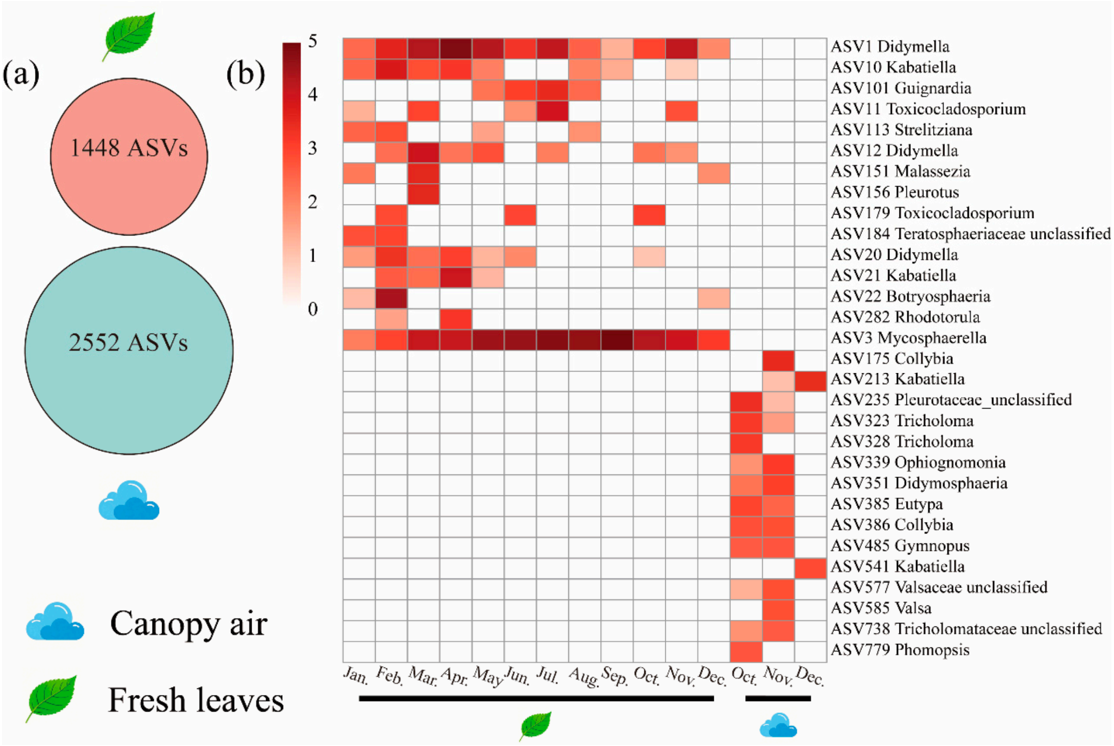

Figure S9. (**a**)Venn diagram of the sharing of endophytic fungi in fresh leaf tissues of A. adenophora throughout the year and canopy airborne fungal communities from October to December at the ASV level. (**b**)Heatmap of the distribution of pathogenic fungi at the ASV level for the top 15 relative abundances of

each of the above communities.

Table S1. Sampling month and environmental factors

**Month**	**Mean monthly temperature (℃)**	**Monthly mean relative humidity (%)**	**Mean monthly wind speed (m/s)**	**Total precipitation (mm)**	**Season**
2021 01	7.98	68.65	4.56	1.78	winter
2021 02	11.51	58.47	4.24	13.72	winter
2021 03	15.70	30.79	5.26	11.18	spring
2021 04	17.93	48.28	4.62	60.20	spring
2020 05	19.78	49.15	4.57	30.23	spring
2020 06	21.87	69.63	4.54	153.67	summer
2020 07	19.81	78.25	3.88	355.60	summer
2020 08	19.89	77.94	2.98	258.57	summer
2020 09	18.54	81.83	3.04	96.52	autumn
2019 10	16.31	75.88	3.43	73.91	autumn
2019 11	13.40	72.49	3.95	8.13	autumn
2019 12	7.92	64.01	3.97	9.65	winter

Table S2. Relative abundance of fungal trophic modes across seasons on the basis of the FUNGuild database

Method	Sample type	Time period	Pa	Sa	Sy
ICM	FL	All	27.74%	51.09%	21.17%
		Spring	17.95%	69.23%	12.82%
		Summer	34.43%	44.26%	21.31%
		Autumn	33.33%	40.74%	25.93%
		Winter	18.18%	63.64%	18.18%
	Air	All	36.91%	47.65%	15.43%
		Spring	24.82%	72.88%	2.30%
		Summer	65.31%	28.23%	6.46%
		Autumn	55.56%	37.20%	7.25%
		Winter	23.08%	12.09%	64.84%
	DL	All	29.24%	46.57%	24.19%
		Spring	9.47%	67.37%	23.16%
		Summer	47.83%	34.78%	17.39%
		Autumn	55.74%	26.23%	18.03%
		Winter	21.33%	44.00%	34.67%
CFM	FL	All	56.30%	11.85%	31.85%
		Spring	47.54%	28.35%	24.11%
		Summer	55.55%	15.29%	29.16%
		Autumn	71.16%	4.73%	24.12%
		Winter	51.96%	2.64%	45.40%
	Air	All	23.82%	66.03%	10.15%
		Spring	35.27%	57.92%	6.81%
		Summer	20.33%	66.73%	12.95%
		Autumn	15.68%	75.33%	8.98%
		Winter	19.80%	68.28%	11.92%
	DL	All	53.06%	33.69%	13.25%
		Spring	48.14%	30.36%	21.50%
		Summer	47.41%	35.07%	17.52%
		Autumn	62.03%	32.32%	5.65%
		Winter	55.48%	36.54%	7.98%

Note: Pa, Sa and Sy represent pathotroph, saprotroph and symbiotroph, respectively. FL and DL represent fresh and dead leaves, respectively.

Table S3. Correlations between fungal community structure and environmental factors analysed via CCA

Method	Sample type	Environmental factor	R2	P
ICM	Air	T	0.4068	0.007**
		RH	0.0713	0.516
		WS	0.0572	0.620
		TP	0.0566	0.602
	FL	T	0.7291	<0.001***
		RH	0.8264	<0.001***
		WS	0.4931	<0.001***
		TP	0.2824	0.015*
	DL	T	0.3713	<0.001***
		RH	0.7212	<0.001***
		WS	0.4180	<0.001***
		TP	0.3287	0.004**
CFM	Air	T	0.281	0.004**
		RH	0.6259	<0.001***
		WS	0.9487	<0.001***
		TP	0.8216	<0.001***
	FL	T	0.2526	0.011*
		RH	0.6876	<0.001***
		WS	0.5137	<0.001***
		TP	0.0737	0.293
	DL	T	0.3332	0.002**
		RH	0.4672	<0.001***
		WS	0.3190	0.001***
		TP	0.3058	0.005**

Table S4. Correlations between pathogenic fungal community structure and environmental factors analysed via CCA and RDA

Method	Sample type	Environmental factor	R2	P
ICM	Air	T	0.7864	<0.001***
		RH	0.6604	0.0290*
		WS	0.2120	0.5872
		TP	0.4691	0.1624
	FL	T	0.1681	0.2024
		RH	0.6428	0.0015**
		WS	0.6251	<0.001***
		TP	0.0291	0.7826
	DL	T	0.6612	<0.001***
		RH	0.5723	<0.001***
		WS	0.8851	<0.001***
		TP	0.4369	0.0015**
CFM	Air	T	0.281	0.0115*
		RH	0.7898	<0.001***
		WS	0.9103	<0.001***
		TP	0.8709	<0.001***
	FL	T	0.3010	0.0035**
		RH	0.4389	<0.001***
		WS	0.3121	0.0025**
		TP	0.0228	0.6757
	DL	T	0.2751	0.0045**
		RH	0.2928	0.0020**
		WS	0.2737	0.0035**
		TP	0.3438	0.0015**

Note: FL and DL represent fresh leaves and dead leaves, respectively. "*" indicates marginally significant (P < 0.05), "**" indicates highly significant (P < 0.01), and "***" indicates extremely significant (P < 0.001).
